# Small EVs From Adipose‐Derived MSCs Modulate Epidermal Barrier and Inflammation Via Sphingosine‐1‐Phosphate Signaling Pathway

**DOI:** 10.1002/jev2.70121

**Published:** 2025-07-22

**Authors:** Kyong‐Oh Shin, Jun Ho Lee, Seungwoo Chae, Karin Goto, Hahyun An, Joan S. Wakefield, Dae Hyun Ha, Healim Lee, Kyojin Lee, Hyunju Lee, Ella Shin, Min Ji Kang, Sinhee Lee, Yoshikazu Uchida, Byong Seung Cho, Kyungho Park

**Affiliations:** ^1^ Department of Food Science and Nutrition, Convergence Program of Material Science for Medicine and Pharmaceutics Hallym University Chuncheon Republic of Korea; ^2^ LaSS Lipid Institute (LLI) LaSS Inc. Chuncheon Republic of Korea; ^3^ ExoCoBio Exosome Institute (EEI) ExoCoBio Inc. Seoul Republic of Korea; ^4^ Department of Dermatology, School of Medicine, University of California San Francisco, Department of Veterans Affairs Medical Center, San Francisco Northern California Institute for Research and Education San Francisco California USA; ^5^ Department of Cosmetic Industry Chungbuk National University Cheongju Republic of Korea

**Keywords:** adipose tissue‐derived mesenchymal stem cells, atopic dermatitis, ceramide metabolic enzyme, lipid, skin, small extracellular vesicles, sphingosine‐1‐phosphate

## Abstract

Epidermal permeability barrier defects are associated with several skin diseases, including atopic dermatitis (AD). Using an AD mouse model, we previously demonstrated that topically administered small extracellular vesicles (sEVs) (prepared following the International Society of Extracellular Vesicles recommendations) from human adipose tissue‐derived mesenchymal stem cells (ASC) ameliorate skin inflammation and normalize barrier function in parallel with increased ceramide (a key barrier lipid) production. To elucidate *how* ASC‐sEVs alleviate these AD skin abnormalities, we characterized lipids and ceramide metabolic enzymes in ASC‐sEVs versus donor ASCs. Our study revealed that free fatty acid, ceramide, and sphingomyelin are enriched in ASC‐sEVs versus donor ASCs, while the synthetic enzymes of ceramide (and acidic sphingomyelinase), and sphingosine‐1‐phosphate (sphingosine kinase) are significantly higher in ASC‐sEVs versus donor ASCs. Conversely, ceramide (ceramidase), and sphingosine‐1‐phosphate hydrolytic enzymes (sphingosine‐1‐phosphate lyase and sphingosine‐1‐phosphate phosphatase) are lower in ASC‐sEVs, suggesting that ceramide and sphingosine‐1‐phosphate levels could elevate in cells that receive ASC‐sEVs. ASC‐sEV‐mediated increases in sphingosine‐1‐phosphate suppress pro‐inflammatory cytokine production in AD‐model human keratinocytes. Additionally, keratinocyte differentiation, which is required for a competent epidermal permeability barrier, was restored in AD‐model human keratinocytes treated with ASC‐sEVs. Taken together, cells that endocytose ASC‐sEVs can normalize epidermal permeability barrier function as well as alleviate inflammation by stimulating a sphingosine‐1‐phosphate signalling pathway.

## Graphical abstract

Ceramide and S1P levels could be elevated in cells that receive ASC‐sEVs. ASC‐sEVs suppress the inflammatory response and normalize differentiation in AD‐model KC via restoration of SPHK1‐mediated S1P synthesis (S1P pathway).

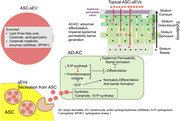

## 1 Introduction

In order for mammals to survive in a terrestrial environment, they must have a competent epidermal permeability barrier. Attenuation of epidermal permeability barrier integrity causes xerosis and skin inflammation via entry of allergens, pathogens and chemicals, as well as excess water evaporation. Epidermal permeability defects also initiate a myriad of adverse skin health consequences (Schmuth et al. 2024). Lipids are an essential component of skin, forming the epidermal permeability barrier in the epidermis’ outermost layer, the stratum corneum, as well as forming various cellular membranes such as the plasma membrane and intracellular organelles (Schmuth et al. 2024). Certain lipids are not only structural components, but also mediators that modulate cellular functions; for example, proliferation, differentiation, cell death, inflammation and tumorigenesis (Fyfe et al. 2023).

**FIGURE 1:**
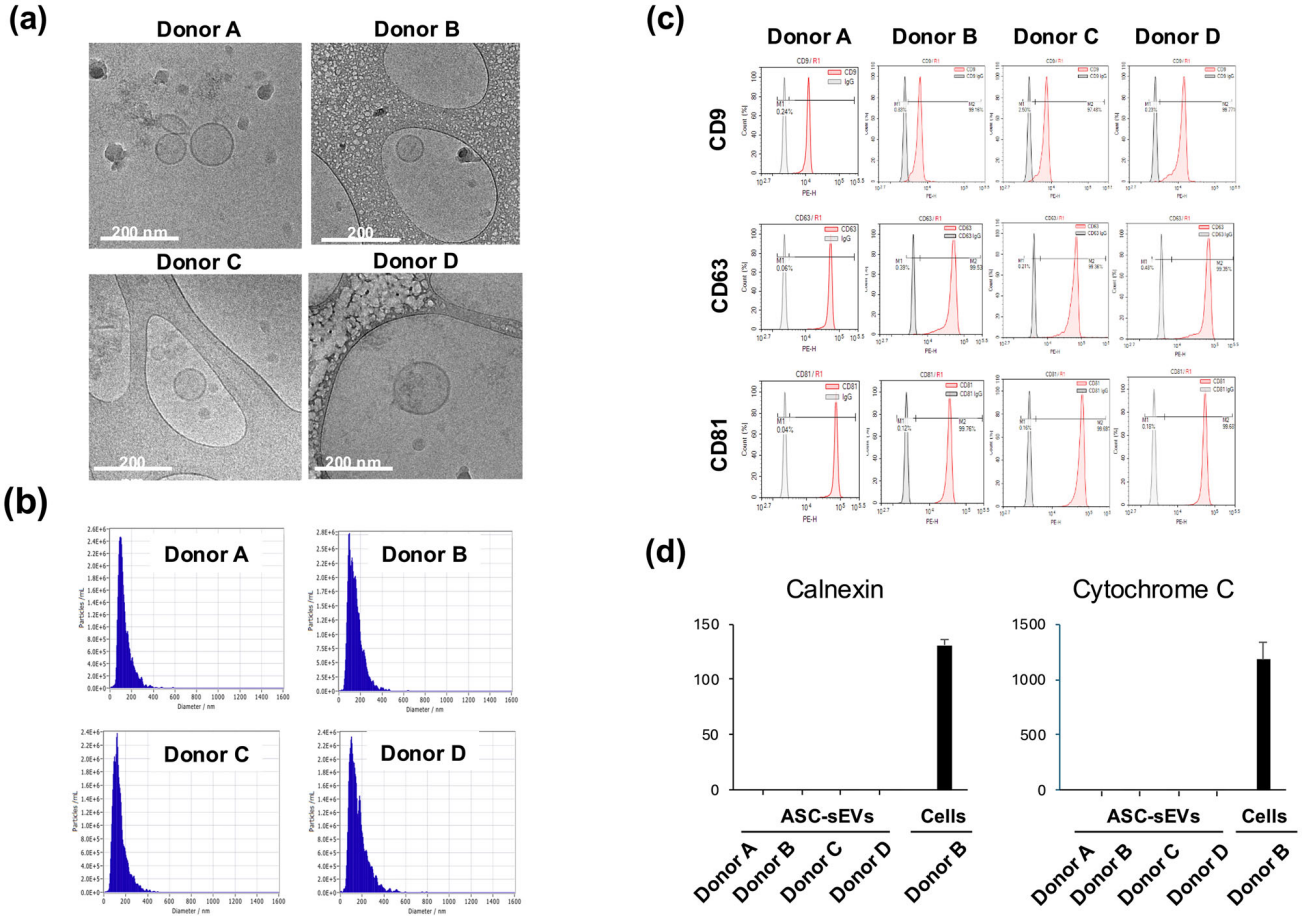
**Characteristics of ASC‐sEVs from four ASC lines**. (a) The morphology of ASC‐sEVs (Donor A, Donor B, Donor C, Donor D) from four ASC lines by cryo‐transmission electron microscopy (cryo‐TEM) analysis. Scale bar = 200 nm. (b) Histogram of particle concentration, size distribution of isolated ASC‐sEVs from four ASC cell banks by nanoparticle tracking analysis (NTA). (c) Exosome positive surface markers (CD9, CD63, CD81) of ASC‐sEVs assessed by flow cytometry. (d) The levels of endoplasmic reticulum (ER) marker calnexin and the mitochondrial marker cytochrome C in ASC‐sEVs. Both calnexin or cytochrome C were at residual levels (<1 ng/mL) in ASC‐sEVs (triplicate assay). See details in Section 2.

Small extracellular vesicles (sEVs) are defined as ‘exosomes’ (prepared according to the International Society of Extracellular Vesicles recommendation) (see details in Section 2, and in Figure 1) in this study. 40–100 nm sized sEVs that carry nucleotides, miRNA, peptides, enzymes and lipids are produced by various cells in the body and are endocytosed into other cells, then affecting various cellular functions (Wang et al. 2020; Yang, Lee, et al. 2021). sEV content varies, depending upon what type of cell it originates from; and sEVs play a pathological role in inflammation, cancer and senescence. In addition to having negative effects on host cells, sEVs can positively enhance wound healing and rejuvenation (Yang, Lee, et al. 2021; Lee, Lötvall, et al. 2023; Lee, Won et al. 2023; Chamberlain et al. 2024). Furthermore, human adipose tissue‐derived mesenchymal stem cell‐derived small extracellular vesicles (ASC‐sEVs) activate neprilysin, an enzyme that degrades the amyloid β (Aβ) peptide (key peptide in Alzheimer's disease development/progression) and has been shown to improve histopathological scoring, suppress disease activity index (DAI), and reduce inflammatory cytokines (TNF‐α, iNOS, IL‐1β, IL‐6) in an inflammatory bowel disease (IBD) animal model. Recently, neprilysin's anti‐virus effects in SARS‐CoV‐2 and H1N1 influenza‐induced acute lung injury models were reported (Wang et al. 2020; Lee, Jeon, et al. 2024).

Atopic dermatitis (AD) is a common inflammatory skin disease associated with epidermal permeability barrier defects (Schmuth et al. 2024). Two mechanisms are implicated in AD pathogenesis; that is, outside (barrier abnormality)‐to‐inside (immune abnormality; i.e., inflammation) and inside (immune abnormality)‐to‐outside (barrier abnormality) (Elias et al. 2008; Hatano and Elias 2023), suggesting that both enhancing epidermal permeability barrier function and suppressing inflammation are therapeutic strategies for AD relief. We earlier demonstrated that topical administration of sEVs derived from ASC improves epidermal permeability barrier integrity and ameliorates inflammation in an AD mouse model (induced by oxazolone); that is, ceramide (a key barrier lipid constituent) levels and type 2 pro‐inflammatory cytokine levels are increased and decreased, respectively, while RNAseq analysis revealed that gene expression associated with epidermal differentiation and lipid production, including ceramide and its metabolites related to epidermal permeability barrier formation, is increased (Shin et al. 2020). In addition to our study, it has been shown that ASC‐sEV administration alleviates inflammation in another type of AD mouse model (Nc/Nga mice) (Cho et al. 2018). Thus, ASC‐sEVs should be useful to treat inflammatory skin diseases associated with compromised epidermal permeability barrier function, such as AD. Yet, the mechanism by which ASC‐sEVs ameliorate AD‐like skin conditions has not been elucidated.

Because our prior study demonstrated that levels of ceramide, a key epidermal permeability barrier constituent, are increased in parallel with ameliorating inflammatory conditions and barrier abnormalities (Shin et al. 2020), we focus here on lipids and ceramide metabolic enzymes in ASC‐sEVs to explore the role of EV‐mediated suppression of inflammation and improvement of barrier integrity in AD. We characterized the profiles of the lipids and the ceramide metabolic enzymes of ASC‐sEVs compared to their donor cells. We then investigated the pathway of ASC‐sEV‐mediated attenuation of inflammatory response using an established Th2 inflammation model of cultured human keratinocytes that mimics AD (Shin et al. 2020).

## 2 Materials and Methods

### 2.1 Kits and Reagents

The kits and reagents used in this study were purchased from the following sources: Minimum Essential Medium (MEM)‐α, foetal bovine serum (FBS), gentamycin, Dulbecco's phosphate buffered saline (DPBS) and trypsin‐ethylenediaminetetraacetic acid (Trypsin‐EDTA) were from Thermo Fisher Scientific; basic fibroblast growth factor (bFGF) was from R&D Systems (Minneapolis, MN, USA); normal immunoglobulin G and phycoerythrin conjugated antibodies for a cluster of differentiation of CD9, CD63 and CD81 were from BD Biosciences (San Jose, CA, USA); the 500‐kDa molecular weight cutoff filter membrane cartridge was from GE Healthcare (Chicago, IL, USA); isopropylalcohol and methanol were from Merck (Darmstadt, Germany); acetonitrile was from Honeywell Burdick & Jackson (Morris Plains, NJ, USA); 16:0‐d31 lyso PC, 17:0‐16:1 PI‐d5, 17:0‐18:1 PI‐d5, 18:0 PA‐d70, 18:1 lyso PI, 18:1 PI(5)P, 22:0(2R‐OH) ceramide, 7α‐Hydroxy‐3‐oxocholest‐4‐enoic acid‐d3, lyso SM (d18:1), sphingosine‐d7, 1,2 16:0/16:0‐DAG, 1,2 16:0/18:1‐DAG, 1,2 16:0/18:2‐DAG, 1,2 18:0/18:1‐DAG, 1,2 18:0/18:2‐DAG, 1,2 18:1/18:1‐DAG, 1,2 18:1/18:2‐DAG, 1,2‐16:0/16:0 DAG, 1,2‐18:0/18:0 DAG, 1,2‐dioleoyl‐rac‐glycerol, 1,3 15/15‐DAG, 1,3 18:2/18:2‐DAG, 1,3‐20:0/20:0‐d5 DAG, 1,3‐diolein, 14:0 coenzyme A, 14:0 lyso PA, 14:0 lyso PE, 14:0 PA, 14:0 PA‐d54, 14:0 PC (DMPC), 14:0 PC‐d9, 14:0 PE, 14:0 PS, 14:0 SM (d18:1/14:0), 14:0‐16:0 PC, 14:0‐18:0 PC, 14:0‐d27 lyso PC, 15:0 lyso PC‐d5, 15:0 lyso PE‐d5, 15:0 lyso PI‐d5, 15:0 lyso PS‐d5, 15:0 SM (d18:1/15:0)‐d9, 15:0‐18:1 PA, 15:0‐18:1‐d7‐PA, 15:0‐18:1‐d7‐PC, 15:0‐18:1‐d7‐PI, 15:0‐18:1‐d7‐PS, 16:0 coenzyme A, 16:0 lyso PA, 16:0 lyso PE, 16:0 lyso PI, 16:0 lyso PS, 16:0 PA, 16:0 PA‐d62, 16:0 PC (DPPC), 16:0 PC‐d9, 16:0 PE 16:0 PI, 16:0 SM (d18:1/16:0), 16:0(2R‐OH) ceramide, 16:0(2S‐OH) ceramide, 16:0(d4) coenzyme A, 16:0/16:0 DAG, 16:0‐14:0 PC, 16:0‐18:0 PC, 16:0‐18:1 PA, 16:0‐18:1 PC (POPC), 16:0‐18:1 PE, 16:0‐18:1 PI, 16:0‐18:1 PI(4)P, 16:0‐18:1 PS (POPS), 16:0‐18:2 PA, 16:0‐18:2 PC, 16:0‐18:2 PC‐d5, 16:0‐18:2 PE, 16:0‐18:2 PS, 16:0‐20:4 PA, 16:0‐20:4 PC, 16:0‐20:4 PE, 16:0‐20:4 PS, 16:0‐22:6 PA, 16:0‐22:6 PC, 16:0‐22:6 PC‐d9, 16:0‐22:6 PE, 16:0‐22:6 PS, 16:0‐d31 ceramide, 16:0‐d31 SM, 16:0‐d31‐18:1 PA, 16:0‐d31‐18:1 PC, 16:1 (Δ9‐Cis) PC, 16:1 (Δ9‐Trans) PC, 16:1 aldehyde‐d5, 16:1 PE, 16:1 SM (d18:1/16:1)‐d9, 17:0 cholesteryl ester, 17:0 coenzyme A, 17:0 lyso PE‐d5, 17:0 lyso PI‐d5, 17:0 lyso PS‐d5, 17:0(2R‐OH) ceramide, 17:0(2S‐OH) ceramide, 17:0‐14:1 PE‐d5, 17:0‐14:1 PI‐d5, 17:0‐14:1 PS‐d5, 17:0‐16:1 PE‐d5, 17:0‐16:1 PS‐d5, 17:0‐18:1 PE‐d5, 17:0‐18:1 PS‐d5, 17:0‐20:3 PE‐d5, 17:0‐20:3 PI‐d5, 17:0‐20:3 PS‐d5, 17:0‐22:4 PE‐d5, 17:0‐22:4 PI‐d5, 17:0‐22:4 PS‐d5, 18:0 coenzyme A, 18:0 lyso PA, 18:0 lyso PE, 18:0 lyso PI, 18:0 lyso PS, 18:0 PA, 18:0 PC (DSPC), 18:0 PC‐d9, 18:0 PE, 18:0 PI, 18:0 PS, 18:0 SM (d18:1/18:0), 18:0(2R‐OH) ceramide, 18:0(2S‐OH) ceramide, 18:0‐14:0 PC, 18:0‐16:0 PC, 18:0‐18:1 PA, 18:0‐18:1 PC, 18:0‐18:1 PE, 18:0‐18:1 PS, 18:0‐18:2 PA, 18:0‐18:2 PC, 18:0‐18:2 PE, 18:0‐18:2 PS, 18:0‐20:4 PA, 18:0‐20:4 PC, 18:0‐20:4 PE, 18:0‐20:4 PI, 18:0‐20:4 PI(3,4,5)P3, 18:0‐20:4 PI(3,5)P2, 18:0‐20:4 PI(4)P, 18:0‐20:4 PI(4,5)P2, 18:0‐20:4 PS, 18:0‐22:6 PA, 18:0‐22:6 PC, 18:0‐22:6 PC‐d9, 18:0‐22:6 PE, 18:0‐22:6 PS, 18:0‐d35 lyso PC, 18:1 (8‐cis) PC 18:1, (Δ9‐cis) PE (DOPE), 18:1 (Δ9‐trans) PE, 18:1 Chol ester, 18:1 lyso PA, 18:1 lyso PE, 18:1 lyso PS, 18:1 PA, 18:1 PI, 18:1 PI(3)P, 18:1 PI(3,4)P2, 18:1 PI(3,4,5)P3, 18:1 PI(3,5)P2, 18:1 PI(4)P, 18:1 PI(4,5)P2, 18:1 PS (DOPS), 18:1 SM (d18:1/18:1(9Z)), 18:1 SM (d18:1/18:1)‐d9, 18:1(11‐cis) PC, 18:1(2R‐OH) ceramide, 18:1(2S‐OH) ceramide, 18:1‐14:0 PC, 18:1‐16:0 PC, 18:1‐18:0 PC, 18:1‐d7 lyso PC, 18:1‐d9 SM, 18:2 (cis) PC (DLPC), 18:2 lyso PA, 18:2 PA, 18:2 PE, 18:2 PS, 18:3 (cis) PC, 18:3 PE, 19:0 Lyso PE‐d5, 19:0 lyso PS‐d5 1‐palmitoyl[13C16]3‐palmitoyl‐glycerol (1,3 [13C16]16:0/16:0‐DAG, 1‐palmitoyl[13C16]3‐oleoyl‐glycerol (1,3 [13C]16:0/18:1DAG), 20:0 coenzyme A, 20:0 PC, 20:0(2R‐OH) ceramide, 20:0(2S‐OH) ceramide, 20:1 (cis) PC, 20:1 SM (d18:1/20:1)‐d9, 20:4 (cis) PC, 20:4 lyso PA, 20:4 lyso PI, 20:4 PA, 20:4 PE, 22:0 coenzyme A, 22:0 PC, 22:0(2S‐OH) ceramide, 22:1 (cis) PC (DEPC), 22:1 SM (d18:1/22:1)‐d9, 22:6 (cis) PC, 22:6 PA, 22:6 PE, 22:6 PS, 24:0 coenzyme A, 24:0 PC, 24:0 SM, 24:0(2R‐OH) ceramide, 24:0(2S‐OH) ceramide, 24:0(d4) coenzyme A, 24:1 (cis) PC, 24:1 SM, 24:1 SM (d18:1/24:1)‐d9, 24:1(2R‐OH) ceramide, 24:1(2S‐OH) ceramide, 26:0 coenzyme A, 26:0‐d4 lyso PC, 28:0 coenzyme A, 3‐Keto sphinganine‐d7 (d18:0, HCl salt), 3β,7α‐dihydroxycholest‐5‐enoic acid‐d3, 3β‐Hydroxy‐7‐oxocholest‐5‐enoic acid‐d3, C13‐dihydroceramide‐d7(d18:0‐d7/13:0), C14 ceramide (d18:1/14:0), C14 dihydroceramide (d18:0/14:0), C15 ceramide‐d7 (d18:1‐d7/15:0), C16 ceramide (d18:1/16:0), C16 ceramide‐d7 (d18:1‐d7/16:0), C16 dihydroceramide (d18:0/16:0), C16 LPA, C16:1 ceramide‐d7 (d18:1‐d7/16:1), C18 ceramide (d18:1/18:0), C18 ceramide‐d7 (d18:1‐d7/18:0), C18 dihydroceramide (d18:0/18:0), C18 LPA, C18:1 ceramide (d18:1/18:1(9Z)), C18:1 ceramide‐d7 (d18:1‐d7/18:1), C18:1 LPA, C20 ceramide (d18:1/20:0), C20:1 ceramide‐d7 (d18:1‐d7/20:1), C22 ceramide (d18:1/22:0), C22:1 ceramide‐d7 (d18:1‐d7/22:1), C24 ceramide (d18:1/24:0), C24 ceramide‐d7 (d18:1‐d7/24:0), C24 dihydroceramide (d18:0/24:0), C24:1 ceramide (d18:1/24:1(15Z)), C24:1 ceramide‐d7 (d18:1‐d7/24:1(15Z)), C24:1 dihydroceramide (d18:0/24:1(15Z)), C26:0 ceramide (d18:1/26:0), cholestenoic acid‐d5, cholesterol 700016P, cholesterol‐d7, d5‐DG ISTD Mix II, DG Internal Standard Mixture—Ultimate SPLASH, lyso SM (dihydro), (d18:0) lyso‐SM‐d9, monoolein, oxidized 18:2 cholesterol(d7), sphinganine (d18:0), sphinganine‐1‐phosphate (d18:0), sphinganine‐1‐phosphate‐d7, sphinganine‐d7, sphinganine‐d7, sphingosine (d18:1), sphingosine‐1‐phosphate (d18:1), sphingosine‐1‐phosphate‐d7, sphingosine‐d7, TG Internal Standard Mixture—Ultimate SPLASH, oleic acid‐d9, and arachidonic acid‐d11 were from Avanti Polar Lipids (Alabaster, Al, USA); albumin (Bovine Serum Fraction V Fatty Acid‐Free), ammonium formate, calcium chloride, DL‐dithiothreitol glyceryl tri(octanoate‐1,2,3,4‐13C4), glyceryl tri(oleate‐1,2,3,7,8‐13C5), glyceryl tridecanoate, glyceryl tridodecanoate, glyceryl trimyristate, glyceryl trioctanoate, glyceryl trioleate, HEPES, lipid standard (mono‐, di‐, & triglyceride mix), L‐serine‐13C3, potassium chloride, protease inhibitor cocktail, pyridoxal 5′‐phosphate hydrate, sodium acetate, sucrose, triton X‐100, oleic acid‐d34, myristic acid, palmitic acid, γ‐linolenic acid, lignoceric acid, formic acid, potassium phosphate monobasic, and potassium phosphate dibasic were from Sigma–Aldrich (St. Louis, MO, USA); and cholesterol, lipid standards: triglyceride mixtures, fatty acids unsaturated kit, palmitic‐d31 acid, stearic acid, oleic acid, linoleic acid, arachidic acid, behenic acid, erucic acid and nervonic acid were from Supelco (Bellefonte, PA, USA).

### 2.2 Cultured Adipose Tissue‐Derived Mesenchymal Stem Cells (ASCs)

Human adipose tissue‐derived mesenchymal stem cells (ASCs) were prepared as we described previously (Shin et al. 2020; Lee et al. 2020; Lee, Jeon, et al. 2024). Briefly, human adipose tissue from a healthy female donor (26 ± 3.2 years) (Table S1) was obtained by ExoCoBio Inc. (Seoul, Korea) with approval of the Institutional Review Board of CHA University Medical Center, Korea (IRB No. CHAMC 2019‐05‐040‐018) and evaluated according to the Korean Ministry of Food and Drug Safety (MFDS) guidelines. ASC (passage four) were cultured with MEM‐α containing 10% FBS, 10 ng/mL bFGF, and 1% gentamycin (Gibco, Grand Island, NY, USA). All cells were cultured in a humidified atmosphere of 5% CO_2_ in air at 37°C. Total cell number, cell viability, and cell size were assessed by an automated cell counter (LUNA‐II Automated Cell Counter, Logos Biosystem, Gyeonggi‐do, Republic of Korea) with trypan blue staining. ASC properties were characterized for cell surface protein expression (positive markers CD29, CD90, CD105, and negative markers CD31, CD45, HLA‐DR) by flow cytometry according to the criteria described by the International Society of Cellular Therapy (Dominici et al. 2006) (Table S1). The quality of ASCs was assessed by tests for sterility, mycoplasma, adventitious viruses (HBV, HCV and HIV) and endotoxin (Table S1).

### 2.3 Isolation of ASC‐sEVs

ASC‐sEVs (ASCE, ASCE is the proprietary trademark of ExoCoBio Inc.) were isolated from the conditioned medium (CM) of ASCs by tangential flow filtration (TFF)‐based ExoSCRT technology, as previously described (Lee et al. 2020; Lee, Jeon, et al. 2024). Briefly, the CM was filtrated through a 0.22‐µm polyethersulfone membrane filter (Merck Millipore, Billerica, MA, USA) to remove non‐EV particles such as cells, cell debris, microvesicles, and apoptotic bodies. The CM was then concentrated by tangential‐flow filtration with a 500 kDa molecular weight cutoff filter membrane cartridge (GE Healthcare, Chicago, IL, USA). The properties of ASC‐sEVs were analysed following the Minimal Information for Studies of Extracellular Vesicles 2018 recommendations from the International Society of Extracellular Vesicles (Thery et al. 2018). The morphology of ASC‐sEVs was analysed by transmission electron microscopy (TEM) analysis (Figure 1a). Histograms of particle concentration and size distribution of ASC‐sEVs were analysed by nanoparticle tracking analysis (NTA) (Figure 1b). The sEV surface protein markers (CD9, CD63 and CD81) of ASC‐sEVs were assessed by flow cytometry (Figure 1c). Additionally, calnexin and cytochrome C (markers of endoplasmic reticulum and mitochondria, respectively) content were assayed by ELISA (calnexin, LSBio, Newark, CA and cytochrome C, Thermo Fisher) in order to assess the purity of EVs; that is, free from cellular contamination (Figure 1d).

### 2.4 Cellular Uptake of ASC‐sEVs

ASC‐sEVs were labelled with PKH67 (Sigma–Aldrich) and subsequently purified using PD MiniTrap G‐25 columns (Cytiva, Marlborough, MA, USA) to remove unincorporated dye. To exclude potential interference from the dye itself, the same labelling and purification procedures were applied to the vehicle control group. HaCaT cells were cultured and replaced in a serum‐free medium prior to treatment with the labelled ASC‐sEVs. After 6 or 24 h of incubation, the cells were fixed with 10% formalin solution (Biosesang, Seoul, Republic of Korea). Fixed cells were then counterstained with PKH26 (Sigma–Aldrich) to visualize the cell membrane and Hoechst 33258 (Sigma–Aldrich) for nuclear staining. Cellular uptake of ASC‐sEVs was assessed using a fluorescence microscopic system (Logos Biosystems, Gyeonggi‐do, Republic of Korea) (Maged et al. 2024).

### 2.5 Lipid Analysis

Total lipids were extracted from ASC‐sEVs and donor cells using the Bligh and Dyer method, with modification, as described previously (Park et al. 2013, 2016). Free fatty acids, glycerides, glycerophospholipid, sphingolipids, sterols and their total 227 lipid species were quantitated using LC‐ESI‐MS/MS (Applied Biosystems 5500 QTRAP, Applied Biosystems), as described previously (Wijesinghe et al. 2010; Park et al. 2013, 2016). Mass spectrometry quantification of lipid species in the sample was conducted by multiple reaction monitoring (MRM) mode. For global lipidomics analyses, full scan MS spectra at 100,000 resolutions (defined at *m*/*z* 400) were collected on a Thermo Scientific LTQ‐Orbitrap Velos mass spectrometer in both positive and negative ionization modes. Scans were collected from *m*/*z* 200 to *m*/*z* 2000. Lipids were identified using the Lipid Mass Spectrum Analysis (LIMSA) v.1.0 software linear fit algorithm with an in‐house database. Internal standards are listed in the Supporting Information.

### 2.6 Enzyme Activity Assay

Ceramide metabolic enzymes; that is, serine palmitoyltransferase (SPT), ceramide synthase (CerS)1, CerS2, CerS3, CerS4, Cer5/6, acidic ceramidase (aCDase), neutral CDase (nCDase) and alkaline CDase (alkCDase), acidic sphingomyelinase (aSMase), neutral sphingomyelinase (nSMase), sphingomyelin deacylase (SM deacylase), sphingosine kinase (SPHK) 1 and SPHK2, sphingosine‐1‐phosphate lyase (S1P lyase) and sphingosine‐1‐phosphate phosphatase (S1P phosphatase), were measured using homogenates of ASC‐sEVs and cells, as described previously with modification (Houben et al. 2006; Park et al. 2016; Shin et al. 2024). Stable isotope‐labelled compounds were used as substrates. After terminating the reaction by adding CHCl_3_: MeOH (2:1, *v/v*), an internal standard was added for an enzyme reaction. End products were determined by LC‐ESI‐MS/MS system. Enzyme activities are expressed as pmol of products per mg protein per min. The protein amount was determined by Pierce BCA protein assay kit (Thermo Fisher Scientific, Waltham, USA). The detailed geological analysis methods are as follows:

#### 2.6.1 Serine Palmitoyl Transferase (SPT)

Assay mixture: 100 mM HEPES (pH 8.0), 5 mM dithiothreitol, 20 µM pyridoxal 5′‐phosphate, 50 µM palmitoyl‐CoA, 2 mM L‐Serine‐^13^C_3_ 2 mM L‐serine, and the protease inhibitor cocktail at 37°C for 20 min. Internal standard: C17‐sphinganine. SPT activity was expressed as pmol (^13^C_3_‐3‐ketosphinganine production) per mg protein per min.

#### 2.6.2 Ceramide Synthases (CerS)

Assay mixture: 20 mM HEPES, pH 7.4, 25 mM KCl, 2 mM MgCl_2_, 0.5 mM DTT, 0.1% fatty acid‐free BSA and 50 µM fatty acid‐CoA (C16:0, C18:0, C20:0, C22:0, C24:0 and C26:0), were incubated with 10 nmol of C17‐sphinganine for 30 min at 37°C. Internal standard: d17:1/C18:0 ceramide. CerS activity was expressed as pmol (d17:0 dihydroceramide production) per mg protein per min.

#### 2.6.3 Ceramidase (CDase)

Assay mixture: 25 mM sodium acetate buffer, pH 4.5 (for aCDase), 50 mM HEPES buffer pH 7.4 with 1 mM CaCl_2_ (for nCDase), 50 mM HEPES buffer pH 9.0 with 1 mM CaCl_2_ for alkCDase, were incubated with d18:1‐d7 sphingosine at 37°C for 2 h. Internal standard: sphinganine‐d7. CDase activity was expressed as pmol (d18:1‐d7 sphingosine production)/mg protein/min.

#### 2.6.4 Sphingomyelinases (SMase)

Assay mixture: 25 mM sodium‐acetate for acidic sphingomyelinase, 0.2% Triton X‐100, pH 5.0, and neutral sphingomyelinase: 20 mM HEPES, 0.2% Triton X‐100, pH 7.4) were incubated with 5 nmol of 16:0‐d31 sphingomyelin at 37°C for 20 min. Internal standard: C13‐dihydroceramide‐d7(d18:0‐d7/13:0) SMase activity was expressed as pmol (16:0‐d31 ceramide) per mg protein per min.

#### 2.6.5 Sphingomyelin Deacylase (SM Deacylase)

Assay mixture: 20 mM sodium acetate buffer, pH 5.0, containing 0.8% Triton X‐100 was incubated with 5 nmol of 16:0‐d31 sphingomyelin at 37°C for 30 min. Internal standard: C17 S1P (d17:1). Sphingomyelin deacylase activity was expressed as pmol (Lyso‐SM‐d9) per mg protein per min.

#### 2.6.6 Sphingosine Kinase (SPHK)

Assay mixture: 20 mM Tris‐HCl buffer (pH 7.4) containing 0.5% Triton X‐100, 5 mM EDTA, 5 mM EGTA, 3 mM β‐mercaptoethanol, 5% glycerol and protease inhibitors for SPHK1. 20 mM Tris (pH 7.4) containing 1 M KCl, 5 mM EDTA, 5 mM EGTA, 3 mM β‐mercaptoethanol, 5% glycerol and protease inhibitors for SPHK2. The reaction mixture was incubated with 0.2 mM sphingosine‐d7 and 0.5 mM ATP at 37°C for 30 min. Internal standard: C17 S1P (d17:1). SPHK activity was expressed as pmol (sphingosine‐1‐phosphate d7 production)/mg protein/min.

#### 2.6.7 Sphingosine‐1‐Phosphate Lyase (S1P Lyase)

Assay mixture: 35 mM potassium phosphate buffer, pH 7.4, 0.6 mM EDTA, 70 mM sucrose, 36 mM sodium fluoride, 70 µM pyridoxal‐5 phosphate, protease inhibitors were incubated with 0.2 mM sphingosine‐1‐phosphate‐d7 at 37°C for 20 min. After the termination of the reaction by adding ethanol and 16:1 aldehyde‐d5, 1 mg/mL dansyl hydrazine solution dissolved in acetonitrile and formic acid was added to reaction mixtures and incubated at 60°C for 30 min. Dansyl hydrazine derivatives were analysed by LC‐MS/MS. Internal standard: 16:1 aldehyde‐d5. S1PL activity was expressed as pmol 16:1 aldehyde‐d7 per mg protein per min.

#### 2.6.8 Sphingosine 1‐Phosphate Phosphatase (SPP)

Assay mixture: 100 mM Tris‐maleate buffer, pH 6.5, 1 mM N‐ethylmaleimide, and protease inhibitors were incubated with 0.5 mM sphingosine‐1‐phosphate‐d7 at 37°C for 30 min. The reaction was terminated by the addition of 0.15 M methanolic KOH. Internal standard: sphinganine‐d7. SPP activity was expressed as pmol (d18:1‐d7 sphingosine production)/mg protein/min.

### 2.7 Human Keratinocytes

Non‐transformed human keratinocytes, HaCaT cells (CLS Cell Lines Service GmbH, Germany), were cultured in DMEM supplemented with 10% FCS, as described in our prior studies (Park et al. 2011). Th2 inflammatory model human keratinocyte, which mimics atopic dermatitis‐like inflammation (AD model‐hKC), was prepared as described previously. Briefly, HaCaT cells were treated with a proinflammatory cytokine cocktail, IFN‐γ and TNF‐α (Lee et al. 2018).

### 2.8 qRT‐PCR

Total RNA was isolated from cell lysates using RNeasy mini kit (Qiagen, Germantown, MD), followed by preparation of cDNA using SensiFAST cDNA synthesis kit (Bioline/Meridian, Taunton, MA, USA). mRNA expression levels were assessed by quantitative real‐time polymerase chain reaction (qRT‐PCR) (Park et al. 2011). Relative mRNA expression levels of Il‐1β, IL‐4, and IL‐31 were assessed by qRT‐PCR, as we described previously (Park et al. 2016). The following primer sets were used: IL‐1β F: 5′‐TCTTCTGGGAAACTCACGGC‐3′ and R: 5′‐CCAGACCTACGCCTGGTTTT‐3’, IL‐4 F: 5′‐CCGTAACAGACATCTTTGCTGCC‐3′ and R: 5′‐GAGTGTCCTTCTCATGGTGGCT, and IL‐31 F: 5′‐ACACCGAGTTGGAGAGCCGTAT‐3′ and R: 5′‐CTGTCCTCAGACCGATGTTCTC‐3′

### 2.9 Statistical Analysis

Results were expressed as the mean ± standard deviation (SD). Statistical analyses were performed using Prism Version 6.0 Software (GraphPad Software, San Diego, CA, USA). Significance between groups was determined by unpaired or unpaired Student's *t*‐test, as indicated in tables and figures. The *p* values were set at <0.01.

## 3 Results

### 3.1 Lipid Profiles of ASC‐sEVs and Their Donor Cells

We studied lipid profiles from four batches of adipose tissue‐derived MSC‐sEVs and from their donor (Thery et al. 2018) (26 ± 3.2 years, BMI 25.1 ± 2.0) (Table S1), produced at a GMP (Good Manufacturing Practices) manufacturing facility (ExoCoBio Inc.). We identified five lipid classes; that is, free fatty acids, glycerides, glycerophospholipid, sphingolipids, sterols, and their total 227 lipid species. Glycerophospholipid is the most common lipid species in both ASC‐sEVs (77.69 ± 3.63% of total lipids) and their donor cells (79.60 ± 4.67% of total lipids), followed by sphingolipids (ASC‐sEVs 12.71 ± 2.81% and donor cells 9.95 ± 3.15%), free fatty acids (ASC‐sEVs 6.04 ± 0.81% and donor cells 4.14 ± 0.84%), diglycerides and triglycerides (ASC‐sEVs 2.92 ± 0.07 and donor cells 3.0 ± 4.67%) and sterols (ASC‐sEVs 0.13 ± 0.02 % and donor cells 0.91 ± 0.23%) (Table 1). Although there is no difference in total lipid yield between Donor A and Donor B, Donor D yield is higher than Donor A, Donor B, and Donor C. The total lipid yield of Donor C is lower than Donor D, Donor A and Donor B (Table S2). However, there are no significant differences in the ratio of each lipid species among the four batches in ASC‐sEVs and in their donor cells (Table 1).

**TABLE 1:** Ratio of lipid to total lipid amounts (%).

	ASC‐sEVs						Donor cells						
	Donor A	Donor B	Donor C	Donor D	Mean	SD	Donor A	Donor B	Donor C	Donor D	Mean	SD	*t*‐test
Total sterol^*^	0.121	0.11	0.127	0.15	0.127	0.017	0.851	0.664	1.22	0.92	0.914	0.231	0.001
FFA	6.036	6.835	6.363	4.922	6.039	0.814	2.888	4.729	4.399	4.535	4.138	0.844	0.02
DG	0.274	0.945	0.952	0.977	0.787	0.342	0.38	1.01	1.031	1.014	0.859	0.319	n.d.
TG	1.647	2.485	2.06	2.346	2.135	0.37	1.752	2.559	2.124	2.358	2.198	0.347	n.d.
PA	0.42	0.575	0.486	0.598	0.52	0.082	2.184	2.493	2.195	2.504	2.344	0.179	0.001
PL^**^	82.755	75.573	74.623	77.802	77.688	3.631	86.518	77.476	76.316	78.073	79.596	4.672	n.d.
SPL^***^	8.747	13.477	15.389	13.206	12.705	2.812	5.428	11.069	12.715	10.596	9.952	3.15	n.d.
NS	2.22	3.018	3.085	3.159	2.871	0.437	1.072	2.091	2.136	2.141	1.86	0.526	0.02
NDS	0.537	0.732	0.622	0.527	0.605	0.095	0.256	0.506	0.429	0.357	0.387	0.106	0.02
SP	0.147	0.201	0.171	0.21	0.183	0.029	0.185	0.222	0.193	0.226	0.206	0.021	n.d.
PC	48.082	36.285	39.567	42.756	41.673	5.024	50.199	37.18	40.43	42.882	42.673	5.534	n.d.
PE	31.967	35.608	32.234	31.838	32.912	1.805	33.406	36.488	32.948	31.94	33.695	1.959	n.d.
PS	2.705	3.68	2.821	3.208	3.104	0.44	2.913	3.809	2.938	3.25	3.228	0.417	n.d.

Abbreviations: DG, diglyceride; FFA, free fatty acid; NDS, ceramide NDS; NS, ceramide NS; PA, phosphatidic acid; PC, phosphatidylcholine; PE, phosphatidylethanoamine; PL, glycerophospholipid; PS, phosphatidylserine; SP, sphingoid base‐1‐phosphate; SPL, sphingolipid; TG, triglyceride; *t*‐test, unpaired Student's *t*‐test.

* Total sterol: cholesterol, cholesterol acylester, 25‐hydroxysterol, 27‐hydroxysterol, 7‐ketocholesterolsterol and 6‐keto‐5α‐hydroxycholesterol.

** Total PL: PC, PE, PS.

*** Total SPL: NS, NDS, sphingomyelin, and sphingoid base and SP.

#### 3.1.1 Free Fatty Acids

**FIGURE 2:**
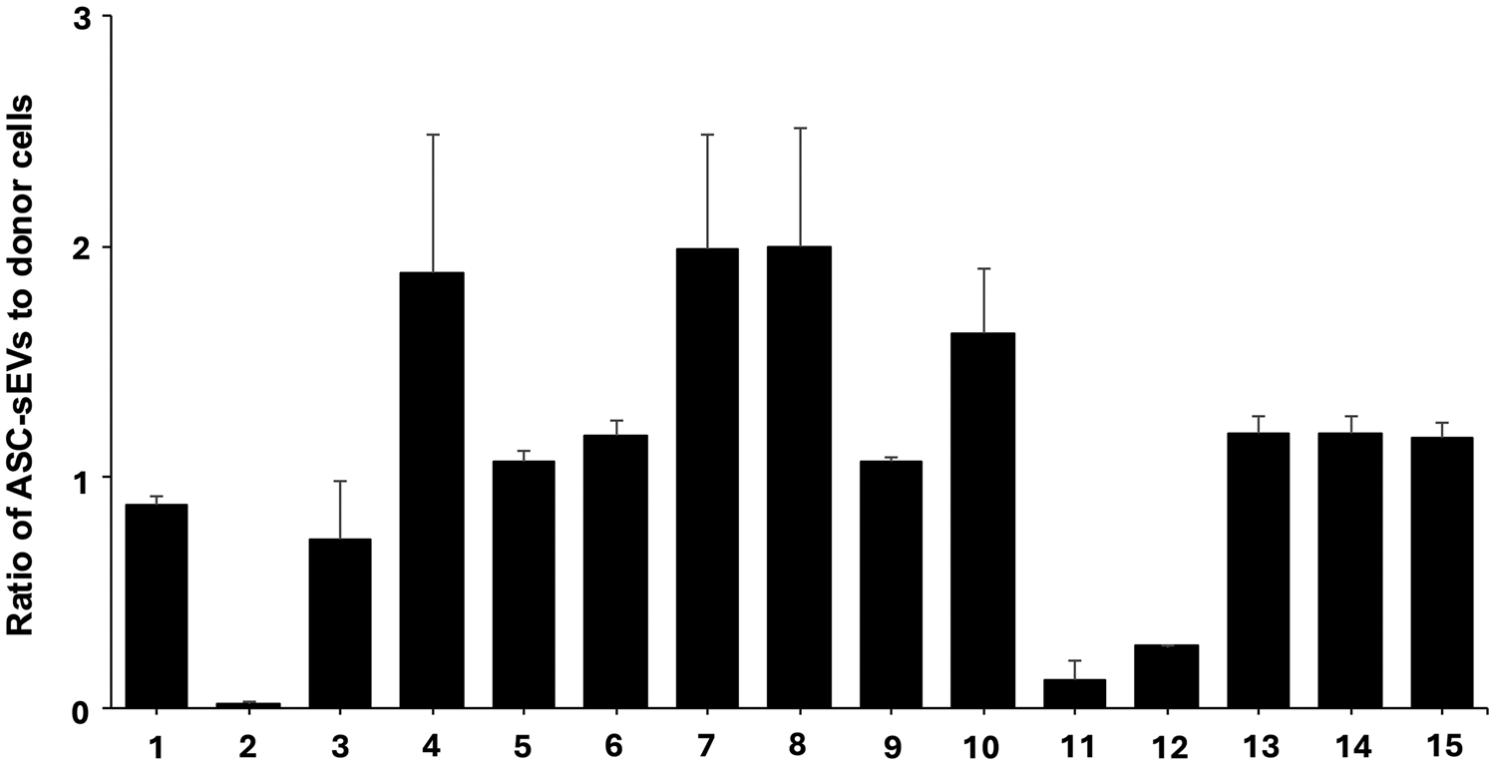
**Lipid profile ratio of ASC‐sEVs compared to donor cells. (**1) Cholesterol, (2) Cholesterol acylester, (3) Other sterols (25‐hydroxysterol, 27‐hydroxysterol, 7‐ketocholesterol, and 6‐keto‐5α‐hydroxycholesterol), (4) Free fatty acid, (5) Diacylglyceride, (6) Triglyceride, (7) Ceramide NS, (8) Ceramide NDS, (9) Sphingoid base. (10) Sphingomyelin, (11) Lysosphingomyelin, (12) Phosphatidic acid, (13) Phosphatidylcholine, (14) Phosphatidylethanoamine, (15) Phosphatidylserine. The ratio was calculated by the lipid content of pmol/mg protein of four subjects derived ASC‐sEVs and donor cells. See details in Section 2. Mean ± SD. *n* = 4, *p* < 0.01 versus donor cells (unpaired *t*‐test).

Carbon chain lengths C14, C16, C16:1, C18, C18:1, C18:2, C18:3, C20, C20:1, C20:4, C20:5, C22, C22:1, C24 and C24:1 fatty acid were detected in both ASC‐sEVs and donor cells. Molecular distribution of fatty acids did not differ between ASC‐sEVs and donor cells, while total free fatty acid levels in ASC‐sEVs were significantly higher than those in donor cells (1.9‐fold, *p* > 0.02) (Figure 2, Tables 1 and S3), suggesting that free fatty acids are efficiently incorporated into sEVs.

#### 3.1.2 Glycerides

There is no significant difference in total diglyceride (DG) content between ASC‐sEVs and donor cells. Yet, the length of the acyl chain and the contents of C14:0/C14:0, C16:0/20:4‐, and C18:0/22:6‐DG in ASC‐sEVs were lower than those in donor cells (Figure 2, Tables 1 and S4). Similar to DG, total triglyceride (TG) content did not differ between ASC‐sEVs and donor cells. The molecular profile of fatty acids was also similar in ASC‐sEVs and donor cells (Figure 2, Tables 1 and S5), suggesting that DG and TG are not enriched in sEVs.

#### 3.1.3 Glycerophospholipids

The three glycerophospholipids, phosphatidylcholine (PC), phosphatidyl ethanolamine (PE), and phosphatidylserine (PS), were contained in the largest lipid species in both ASC‐sEVs and donor cells (Figure 2, Tables 1 and S6–S8). Total phosphatidic acid (PA) levels in ASC‐sEVs were significantly lower compared with donor cells (0.27‐fold donor, *p* < 0.01). However, the contents of PA containing polyunsaturated acids 20:4 and 22:6 were significantly higher (5.5‐fold, *p* < 0.01) than those of donor cells (Figure 2, Tables 1 and S9). These results demonstrate that PA containing polyunsaturated acid are preferentially incorporated into sEVs.

#### 3.1.4 Sterols

Cholesterol, cholesterol acyl ester, hydroxysterol and ketocholesterol were all contained in both ASC‐sEVs and donor cells. Although cholesterol levels were similar in ASC‐sEVs and donor cells, sterol fatty acid ester, sterol, 25‐hydroxysterol, 27‐hydroxysterol, 7‐ketocholesterol and 6‐keto‐5α‐hydroxycholesterol levels in ASC‐sEVs were significantly lower compared with donor cells (*p* < 0.01) (Figure 2, Tables 1 and S10), suggesting that, similar to DG and TG, sterols are not enriched in sEVs.

#### 3.1.5 Sphingolipids

Ceramide, sphingoid bases, sphingoid base 1‐phosphate, sphingomyelin and lyso‐sphingomyelin profiles are characterized below:

##### 3.1.5.1 Ceramide

Since N‐nonhydroxy fatty acyl sphingosine (NS) is a major lipid species in adipocytes, NS and its precursor, N‐nonhydroxy fatty acyl dihydrosphingosine (NDS) were measured. Total NS content was significantly higher (2.0‐fold, *p* < 0.01) in ASC‐sEVs compared with donor cells (Figure 2, Tables 1 and S11). All ceramide NS species, C14‐, C16‐, C18‐, C20‐, C22‐, C24:1‐, C24‐, C26:1‐ and C26‐Ceramide NS, were increased in ASC‐sEVs compared with donor cells. Similar to ceramide NS, ceramide NDS content was significantly higher in ASC‐sEVs compared with donor cells (2.0‐fold, *p* < 0.01) (Figure 2, Tables 1 and S12). These results demonstrate that NS and NDS are enriched in sEVs.

##### 3.1.5.2 Sphingomyelin (SM)

SM species containing C14‐, C16‐, C18‐, C20‐, C22‐, C24:1‐, C24‐, C26:1‐, and C26 amide‐linked fatty acid were present in both ASC‐sEVs and donor cells. SM content in ASC‐sEVs was significantly higher than that in donor cells (1.8‐fold), albeit not statistically significant. Lysosphingomyelin and lysodihydrosphingomyelin levels were significantly lower in ASC‐sEVs compared with donor cells (lysosphingomyelin 8.7%, and dihydrosphingomyelin 1.7 % of donor cells, *p* < 0.01) (Figure 2, Tables 1 and S13). These results demonstrate that SM, but not lysosphingomyelin or lysodihydrosphingomyelin, are enriched in sEVs.

##### 3.1.5.3 Sphingoid Bases and Sphingoid Base‐1‐Phosphates

The content of sphingoid bases (sphingosine and dihydrosphingosine) and sphingoid base‐1‐phosphates (sphingosine‐1‐phosphate and dihydrosphingosine‐1‐phosphate) did not differ between ASC‐sEVs and donor cells (Figure 2, Tables 1 and S14).

In summary, these lipid analyses suggest that in addition to ceramide, free fatty acid and sphingomyelin (all precursors of ceramide in de novo and salvage pathways to ceramide syntheses), endocytosed‐ASC‐sEVs could also increase ceramide levels.

### 3.2 Enzyme Activity of Sphingolipids

**FIGURE 3:**
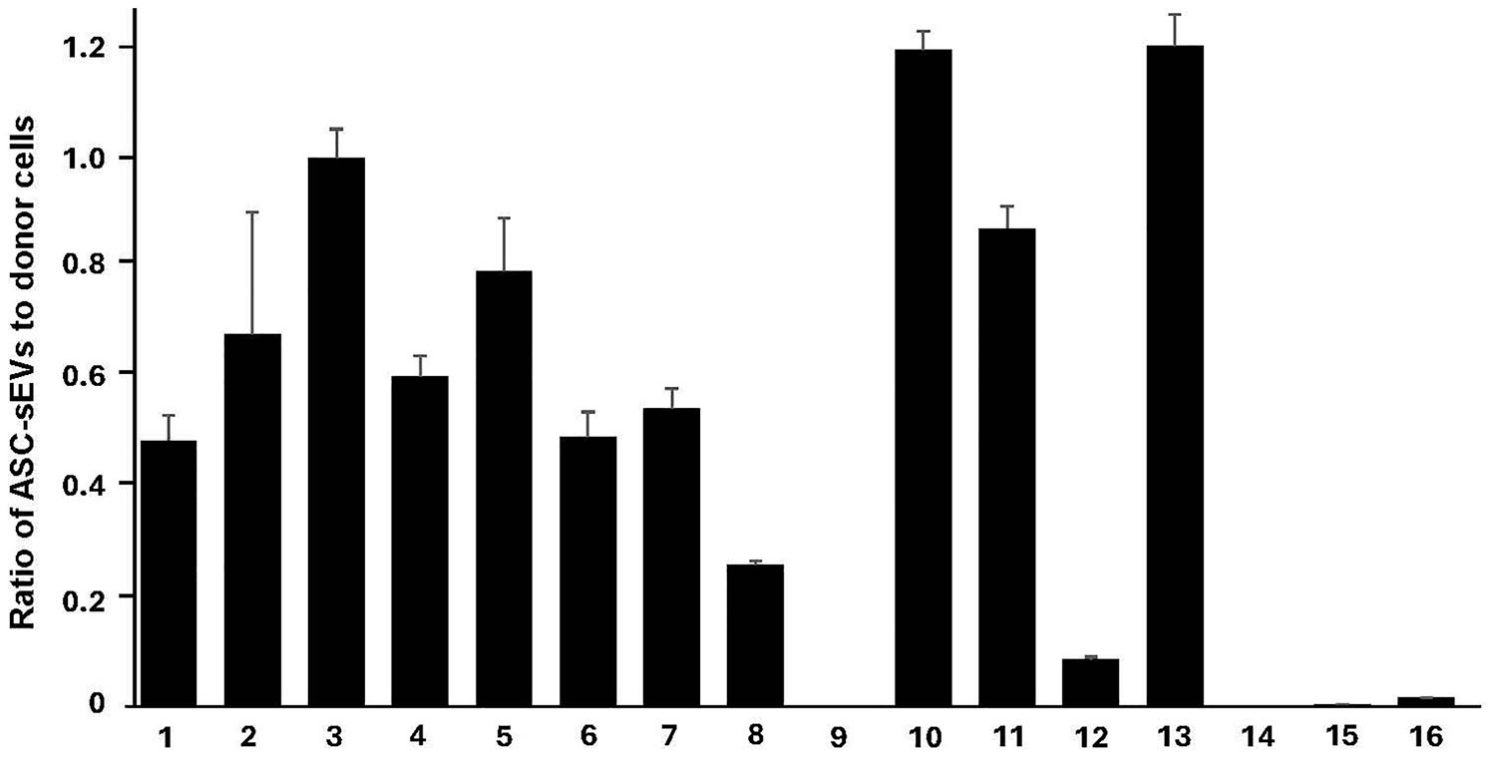
**Ceramide metabolic enzyme activities ratio of ASC‐sEVs compared to donor cells. (**1) Serine palmitoyltransferase (SPT), (2) Ceramide synthase (CerS)1, (3) CerS2, (4) CerS3, (5) CerS4, (6) Cer5/6, (7) Acidic ceramidase (aCDase), (8) Neutral CDase (nCDase), (9) Alkaline CDase, (10) Acidic sphingomyelinase (aSMase), (11) Neutral sphingomyelinase (nSMase), (12) Sphingomyelin deacylase (SM deacylase), (13) Sphingosine Kinase (SPHK) 1, (14) SPHK2, (15) Sphingosine‐1‐phosphate lyase (S1P lyase), (16) Sphingosine‐1‐phosphate phosphatase (S1P phosphatase). See details in Section 2. Mean ± SD. *n* = 4, *p* < 0.01 versus donor cells (unpaired *t*‐test).

Similar to ASC‐sEV and donor cell lipid contents, there are variations in enzyme activities among the four batches of ASC‐sEVs and donor cells (Donor A, Donor B, Donor C, Donor D), but the ratio of each enzyme activity is not significantly different among these four batches (Figure 3, Table 2).

**TABLE 2:** Ceramide metabolic enzyme activities: Ratio of ASC‐Exosome to donor cell

	Donor A	Donor B	Donor C	Donor D	Mean	SD	*t‐test*
SPT	0.431	0.540	0.497	0.467	0.484	0.040	0.01
CerS1	0.996	0.984	0.515	0.515	0.753	0.238	n.s.
CerS2	1.000	1.000	1.000	1.000	1.000	0.000	n.s.
CerS3	0.598	0.613	0.639	0.548	0.600	0.033	0.01
CerS4	0.785	0.691	0.922	0.755	0.788	0.084	0.02
CerS5/CerS6	0.519	0.552	0.448	0.470	0.497	0.041	0.001
Neutral CDase	0.498	0.512	0.594	0.529	0.533	0.037	0.001
xAcidic CDase	0.262	0.249	0.268	0.252	0.258	0.008	0.001
Alkaline CDase	0.000	0.000	0.000	0.000	0.000	0.000	0.001
Acidic SMase	1.214	1.240	1.179	1.160	1.198	0.031	0.01
Neutral SMase	0.875	0.907	0.813	0.879	0.869	0.036	0.01
SM deacylase	0.083	0.092	0.084	0.082	0.088	0.004	0.001
SPHK1	1.202	1.126	1.267	1.205	1.200	0.058	0.01
SPHK2	0.000	0.000	0.000	0.000	0.000	0.000	0.001
S1P lyase	0.004	0.004	0.003	0.003	0.003	0.000	0.001
S1P Phosphatase	0.016	0.016	0.017	0.016	0.016	0.000	0.001

Abbreviations: CDase, ceramidase; CerS, ceramide synthase; SMase, sphingomyelinase; SPHK, sphingosine kinase; SPT, serine palmitoyltransferase; *t*‐test, unpaired Student's *t*‐test. n.s. not statically significant.

#### 3.2.1 Serine Palmitoyl Transferase

Serine palmitoyltransferase (SPT) is the first‐step and rate‐limiting enzyme for de novo ceramide synthesis. Although SPT activity in ASC‐sEVs was significantly lower (0.48‐fold, *p* < 0.01) than that in donor cells (Figure 3, Tables 2 and S15), the SPT activity in ASC‐sEVs was equivalent to that of cultured normal human keratinocytes (37.54 pmol/mg).

#### 3.2.2 Ceramide Synthase

Ceramide synthase (CerS) synthesizes ceramide from dihydrosphingosine and fatty acyl‐CoA. Six types of CerS have been identified in mammalian cells. Each CerS has a substrate specificity (Mizutani et al. 2009); that is, CerS1, C18 fatty acids; CerS2, C22/C24; CerS3 ≥ C26 fatty acids; C18/C20; CerS4, C18 and C20; and CerS5/CerS6, C14 and C16. There were no significant differences in CerS1, CerS2 and CerS4 activities in ASC‐sEVs and donor cells, while CerS3 and CerS5/6 activities in ASC‐sEVs were significantly lower than those in donor cells (CerS3, 0.6‐fold, *p* < 0.01 and CerS5/6, 0.41‐fold, *p* < 0.01) (Figure 3, Tables 2 and S15). However, the activities of these six CerS isoforms in ASC‐sEVs are almost equivalent in human keratinocytes.

Although neither SPT and CerS are enriched in ASC‐sEVs, these enzyme levels are almost equivalent to levels in human keratinocytes, suggesting that because keratinocytes have a potent ability to synthesize large amounts of ceramide (Uchida and Park 2021), ceramide can also be sufficiently synthesized in cells endocytosed with ASC‐sEVs.

#### 3.2.3 Ceramidases

Ceramidase (CDase) hydrolyses ceramide to sphingoid bases and fatty acids, so neutral‐, acidic‐ and alkaline pH optimum (acidic, neutral and alkaline ceramidase) catalytic activities were measured. Alkaline CDase activity was at neglectable levels in both ASC‐sEVs and cultured normal human keratinocytes. Both acidic and neutral CDase activities in ASC‐sEVs were significantly lower compared with donor cells (acidic CDase, 0.26‐fold, *p* < 0.01 and neutral CDase, 0.58‐fold, *p* < 0.01) (Figure 3, Tables 2 and S15). But these acidic and neutral CDase activities in ASC‐sEVs were equivalent to human keratinocyte activities (acidic and neutral ceramidase 33.02 and 63.54 pmol/mg protein, respectively).

#### 3.2.4 Sphingomyelinase

Sphingomyelinase (SMase) hydrolyses sphingomyelin to generate ceramide and phosphorylcholine. There was no difference in neutral‐pH optimal SMase activities among ASC‐sEVs and donor cells (Figure 3, Tables 2 and S15), while acidic‐pH optimal SMase activities were significantly higher in ASC‐sEVs compared with donor cells and human keratinocytes (acidic and neutral SMase 83.56 and 35.72 pmol/mg protein, respectively). Acidic SMase produces ceramide via a salvage pathway. Thus, ceramide production can be increased in cells endocytosed with ASC‐sEVs.

#### 3.2.5 Sphingomyelin Deacylase

Sphingomyelin deacylase (SM deacylase) converts sphingomyelin to sphingosylphosphorylcholine. This enzyme activity is elevated in AD patient skin (Hara et al. 2000; Teranishi et al. 2020). SM deacylase activity in ASC‐sEVs was significantly lower than those in donor cells (0.09‐fold, *p* < 0.01) (Figure 3, Tables 2 and S15). These results suggest that SM can be preferentially hydrolysed to ceramide, but not to sphingoid bases in cells endocytosed with ASC‐sEVs.

#### 3.2.6 Sphingosine Kinase

Sphingosine kinase (SPHK) synthesizes S1P from sphingosine. The SPHK1 activity of ASC‐sEVs was significantly higher than that in donor cells (1.21‐fold, *p* < 0.01) and in keratinocytes (2.72 pmole/mg protein) (2.09‐fold, *p* < 0.01) (Figure 3, Tables 2 and S15).

#### 3.2.7 Sphingosine‐1‐Phosphate Lyase

Sphingosine‐1‐phosphate lyase (S1P lyase) hydrolyses S1P to generate hexadecenal and phosphoethanolamine, which becomes a precursor to synthesize phosphatidylethanolamine. S1P lyase activity is elevated in AD patient skin (Seo et al. 2006; Wood et al. 2009; Baumer et al. 2011). The S1P lyase activities in ASC‐sEVs were significantly lower than those in donor cells (0.003‐fold of donor cells, *p* < 0.01) (Figure 3, Tables 2 and S15) and in keratinocytes (32.41 pmole/mg protein).

#### 3.2.8 Sphingosine‐1‐Phosphate Phosphatase

Sphingosine‐1‐phosphate phosphatase (S1P phosphatase) dephosphorylates S1P to generate sphingosine. S1P phosphatase activities in ASC‐sEVs were significantly lower than those in donor cells (0.02‐fold, *p* < 0.01) (Figure 3, Tables 2 and S15) and in keratinocytes (73.52 pmole/mg protein).

Together with our observations regarding sphingosine kinase, sphingosine‐1‐phosphate lyase and sphingosine‐1‐phosphate phosphatase, these results suggest that generated S1P is not easily hydrolysed in cells endocytosed with ASC‐sEVs.

In summary, acidic sphingomyelinase, CerS and SPHK1 are enriched in ASC‐sEVs. Although SPT is not enriched in ASC‐sEVs, SPT levels are equivalent to those found in keratinocytes, because in addition to ceramide, free fatty acid and sphingomyelin enriched in ASC‐sEVs as above (Figure 1), ceramide levels can be increased in cells that endocytose ASC‐sEVs. Moreover, since ceramide and S1P hydrolytic enzyme (ceramidase, S1P lyase and S1P phosphatase) activities are low in ASC‐sEVs versus donor cells, ceramide and S1P levels could be increased in cells endocytosed with ASC‐sEVs.

### 3.3 ASC‐sEVs Are Endocytosed Into Human Keratinocytes

**FIGURE 4:**
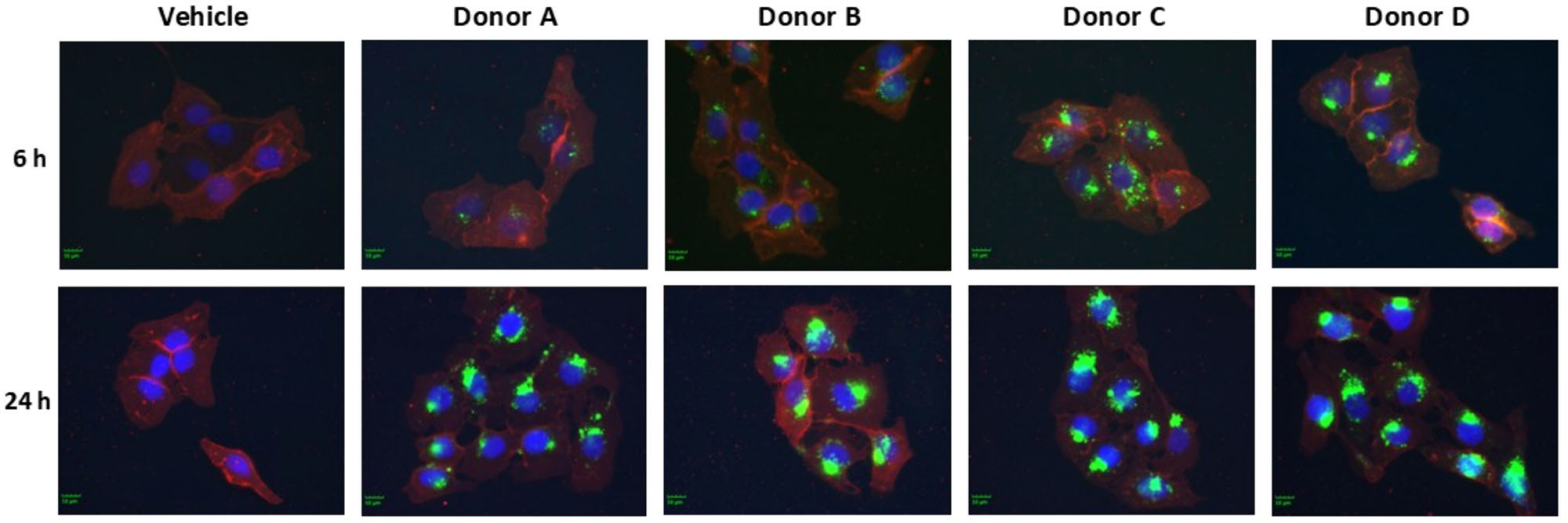
**Incorporation of ASC‐sEVs into human keratinocytes**. HaCaT cells were cultured with PKH67‐labeled ASC‐sEVs for 4 or 24 h. Green fluorescence indicates PKH67‐labeled ASC‐sEVs. Incorporation of PKH67‐labeled ASC‐sEVs into cells was observed using fluorescent microscopy. Scale bar = 10 µm.

Our prior studies demonstrated that topical ASC‐sEVs ameliorate AD‐like inflammation and increase ceramide, S1P and SPHK levels. Prior to conducting our study on *how* ASC‐sEVs suppress inflammation in AD using cultured human keratinocytes, we first verified incorporation of ASC‐sEVs into cells using PKH67 (green fluorescent‐labelled) ASC‐sEVs. Fluorescent cells were observed in cells cultured with fluorescent‐labelled ASC‐sEVs for 6 h (Figure 4). Fluorescence intensities were increased in the cells after fluorescent‐labelled ASC‐sEVs were incubated for 24 h (Figure 4). These results confirmed that keratinocytes were endocytosed with ASC‐sEVs.

### 3.4 ASC‐sEVs Suppress Th2 Cytokine Synthesis by Restoring S1P Production

**FIGURE 5:**
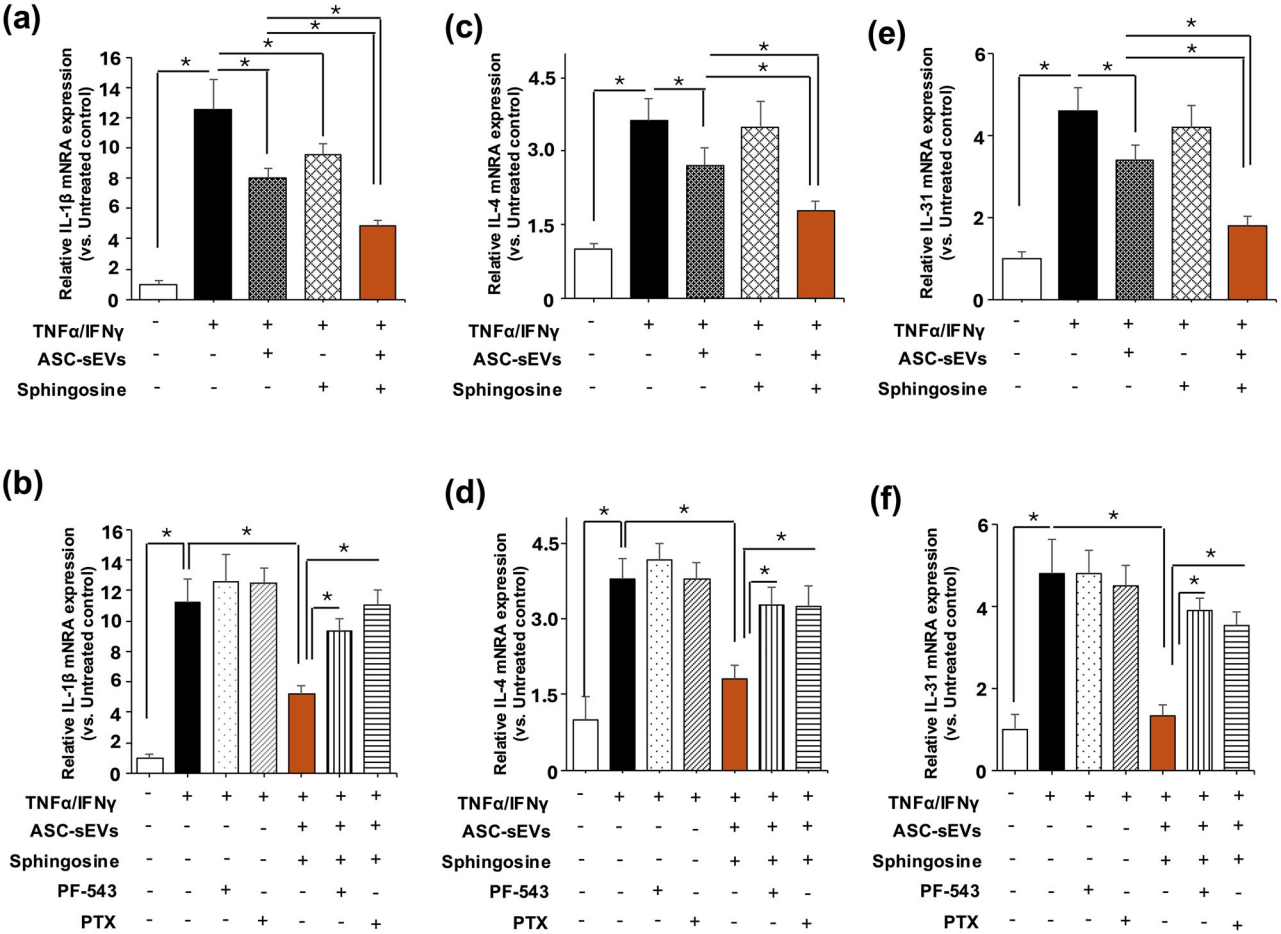
**ASC‐sEVs restore suppressions of Th2 inflammation in atopic dermatitis model of human keratinocytes**. HaCaT cells were cultured with TNF‐α and IFN‐γ (10 ng/mL, each) for 24 h followed by incubation with or without SPHK1 inhibitor, PF‐543, or a pan S1P receptor, pertussis toxin inhibitor, and ASC‐sEVs (3.0E + 10 particles) in DMEM containing 2% FCS for 24 h. IL‐1β, IL‐4 and IL‐31 mRNA levels were assessed by qRT‐PCR. See details in Section 2. Mean ± SD. *n* = 4, *p* < 0.01 versus non‐atopic dermatitis hKC (unpaired *t*‐test).

Next, we investigated the role of S1P in the suppression of inflammation in AD, using an established Th2 inflammatory human keratinocyte (Yang, Kim, et al. 2021), which mimics AD‐like inflammation (AD model‐hKC). Consistent with in vivo AD skin (Baumer et al. 2011), both SPHK1 activity and S1P levels were decreased in AD model‐hKC (SPHK1 activity: 0.63‐fold and S1P: 0.60‐fold), while SPHK2 activity was not altered (Figure 5a,b). SPHK1 activities and S1P content were modestly restored in AD model‐hKC treated with ASC‐sEVs (Figure 5a,b). To further confirm the potential to increase S1P in hKC by addition of sphingosine, a precursor of S1P. As expected, S1P levels were significantly increased in AD model‐hKC co‐incubated with both ASC‐sEVs and a precursor of S1P, sphingosine (S1P: 2.1‐fold) (Figure 5c).

**FIGURE 6:**
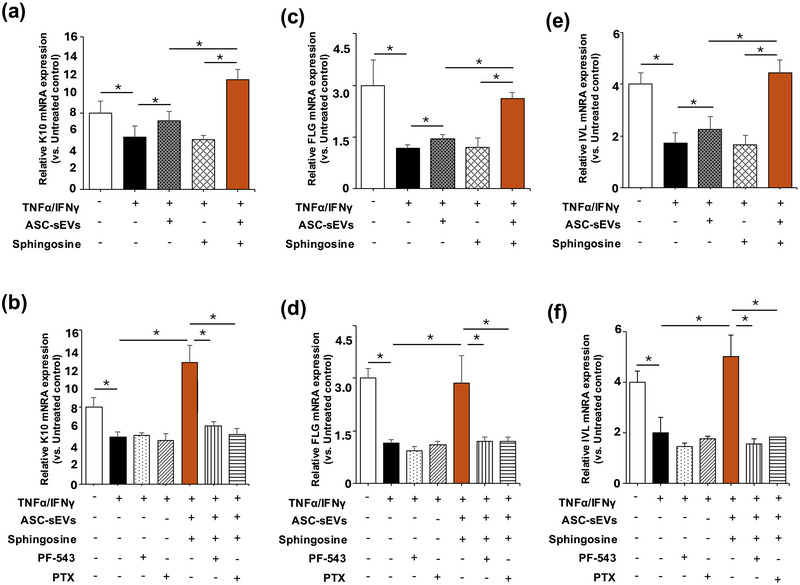
**ASC‐sEVs restore keratinocyte differentiation in atopic dermatitis model‐human keratinocytes**. HaCaT cells were incubated with an inhibitor of SPHK1, PF‐543, or a pan S1P receptor inhibitor, pertussis toxin, in DMEM containing 2% FCS. Keratin 10, involucrin, and filaggrin mRNA levels were assessed by qRT‐PCR. Mean ± SD. *n* = 4, *p* < 0.01 versus non‐atopic dermatitis hKC (unpaired *t*‐test).

**FIGURE 7:**
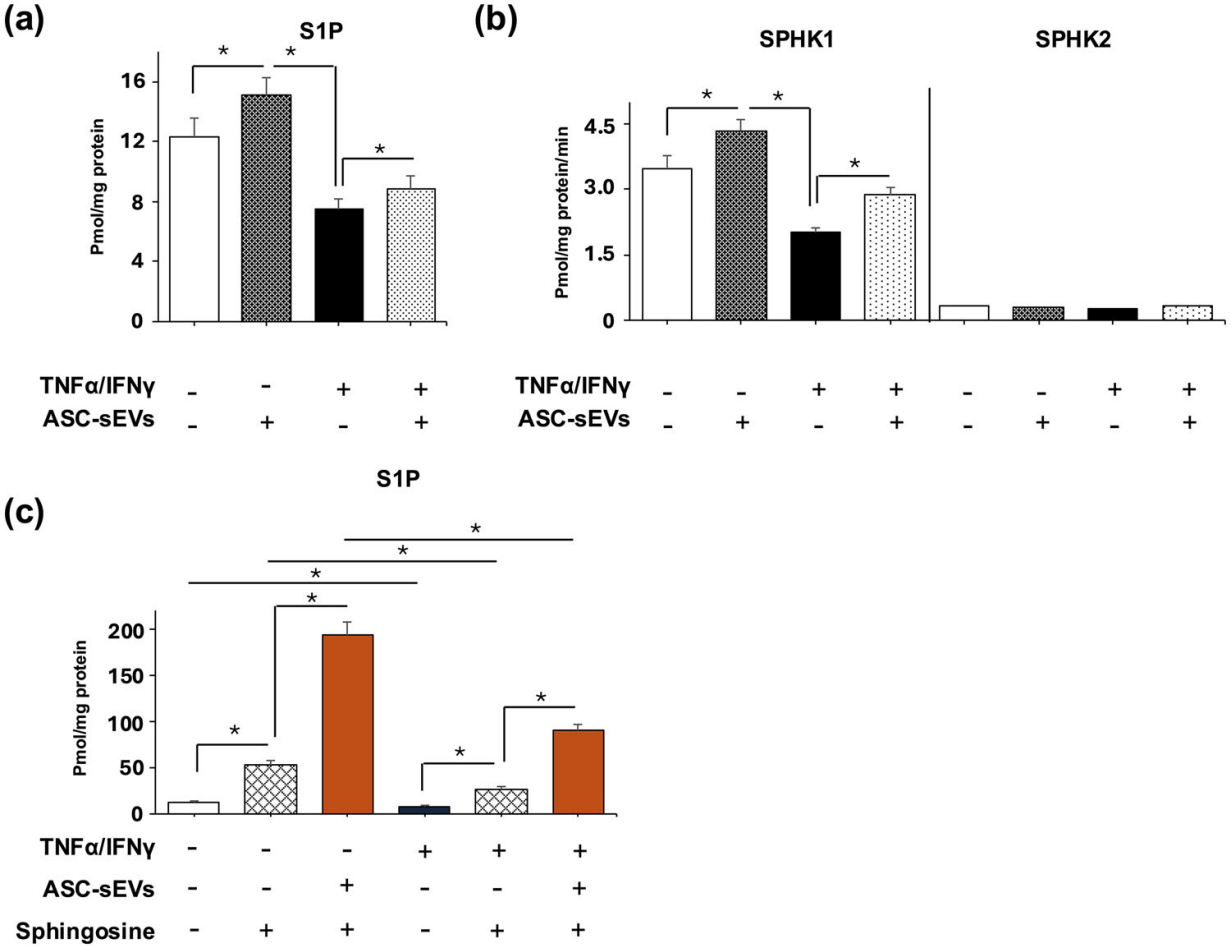
**ASC‐sEVs restore SPHK1 activity and S1P production in atopic dermatitis model‐human keratinocytes**. S1P content (a) and (c), and SPHK1 activity (b). HaCaT cells were cultured with TNF‐α and IFN‐γ (10 ng/mL, each) for 24 h, followed by incubating with or without sphingosine (5 μuM) and ASC‐sEVs (3.0E + 10 particles) in DMEM containing 2% FCS for 24 h. S1P content and SPHK1 activities were measured. Sphingosine, a substrate of SPHK, was added to enhance SPHK activity. See details in Section 2. Mean ± SD. *n* = 4, *p* < 0.01 versus non‐atopic dermatitis KC (unpaired *t*‐test).

We then assessed the effect of ASC‐sEVs on the suppression of proinflammatory cytokines. It is already known that IL‐1β enhances the Th2 inflammatory response (Caucheteux et al. 2016), as well as Th2 cytokine, IL‐1β and Th2 cytokine (IL‐4 and IL‐31) mRNA production. These Th2 cytokine levels were significantly elevated in AD model‐hKC (Figure 6), while increases in Th2 cytokine production were significantly suppressed in cell coincubation with ASC‐sEVs and sphingosine. Yet, the inhibitory effects of ASC‐sEVs‐mediated suppression of Th2 cytokines were attenuated in cells treated with a specific pharmacological inhibitor of SPHK1, PF‐543 (Figure 6). Finally, we investigated how S1P suppresses Th2 cytokine production. Blockade of the S1P receptor by a pan‐S1P receptor inhibitor, pertussis toxin (PTX) (Camerer et al. 2009), significantly diminished ASC‐sEV‐mediated and sphingosine‐mediated suppression of IL‐1β, IL‐4 and IL‐13 (Figure 6). We also found that mRNA levels of keratin 10, filaggrin and involucrin, which are increased during keratinocyte differentiation, are significantly decreased in AD model‐hKC (Figure 7). But differentiation function was restored in keratinocytes incubated with ASC‐sEVs, while SPHK1 inhibition diminished the restoration effect of ASC‐sEVs on keratinocyte differentiation (Figure 7). Th2 cytokines suppressed keratinocyte differentiation (Howell et al. 2008). However, it was notable that mRNA levels of keratin 10, filaggrin, and involucrin in the inhibition of SPHK1 are lower than those in control cells. Hence, S1P deficiency and Th2 cytokines could additively attenuate keratinocyte differentiation.

In summary, ASC‐sEVs can increase S1P levels in AD‐model keratinocytes, followed by S1P receptor activation that suppresses Th2 cytokine production and restores keratinocyte differentiation.

## 4 Discussion

Prior studies investigating various tissue‐derived mesenchymal stem cells have provided evidence that adipose tissue obtained from female donors exhibits significantly better performance compared to that from male donors. Specifically, female‐derived adipose tissue demonstrates higher enhancement of cell proliferation, more effective immune regulatory functions, and greater stability in maintaining cellular senescence homeostasis (Maged et al. 2024). Hence, sEVs from female adipose tissue‐derived mesenchymal stem cells were used in our study. The exact reasons why female donors exhibit significantly better performance versus male donors have not been elucidated. There are a few possibilities about the gender difference; for example, males carry one different chromosome than females, which theoretically could translate proteins that are not present in women that affect cellular function; and recent studies demonstrate that sex chromosomes affect resilience in health (Maged et al. 2024), suggesting that the total number of X chromosomes and silent X chromosomes affect cellular functions (Davis et al. 2019; Gordon and Hubbard 2019; Gadek et al. 2025).

We analysed the profiles of five classes of lipids, showing a total of 227 species, and a total of 16 ceramide metabolic enzymes in four ASC‐sEVs and their donor cells (derived from healthy female subjects). The profiles of both lipids and enzymes did not differ among the four donors, suggesting that the ASC‐sEVs containing the similar lipids and ceramide metabolic enzymes can be reproducibly produced from adipocytes derived from a healthy female, age 26 ± 3.2 years (BMI 25.1 ± 2.0).

Five PA species (16:0/18:2‐, 16:0/20:4‐, 18:0/18:2‐, 18:0/20:4‐ and 18:0/22:6‐PA) were detected in ASC‐sEVs, but not in donor cells. Thus, these PA lipids can be used as markers of ASC‐sEVs for a quality control test standard in GMP production.

Free fatty acid (utilized for de novo ceramide synthesis), ceramides (NS and NDS), sphingomyelin, acidic SMase (which synthesizes ceramide via salvage pathway) and SPHK1 (which synthesizes S1P) are enriched in ASC‐sEVs compared to donor cells. In addition, SPT (a critical, late limiting enzyme of de novo ceramide synthesis in ASC‐sEVs) activity is equivalent to that in human KCs. Hence, ceramide levels are increased by both de novo (initiated by SPT) and salvage (conversion of sphingomyelin to ceramide by SMase) pathways. Moreover, ceramidase, sphingomyelin deacylase, S1P lyase and S1P phosphatase activities are significantly lower in cells treated with ASC‐sEVs versus donor cells. Thus, the metabolic pathway of ceramide‐to‐sphingosine by sphingomyelinase and ceramidase, and then sphingosine‐to‐S1P by SPHK1 should be activated, while the hydrolytic pathway of ceramide and S1P by ceramidase and S1P lyase/S1P phosphatase, respectively, is not activated—thus, high ceramide and S1P levels can be sustained in cells that endocytose ASC‐sEVs.

Skin inflammation of the Nc/Nga AD mouse model was suppressed by ASC‐sEVs treatment (subcutaneous or intravenous injection) (Cho et al. 2018). In addition, we demonstrated that topically applied ASC‐sEVs ameliorate skin symptoms, including inflammation and epidermal permeability barrier abnormalities in AD‐model mice initiated by oxazolone (Shin et al. 2020). Increases in S1P lyase expression and S1P deficiency were also evident in canine AD skin (Wood et al. 2009; Baumer et al. 2011). We found that Th2 cytokine expression is increased and conversely S1P level and SPHK1 activity are decreased in AD‐like in vitro model human keratinocytes, while increases in Th2 cytokine expression were suppressed and decreased SPHK1 and S1P expression was restored in keratinocytes incubated with ASC‐sEVs. Moreover, co‐incubation with an inhibitor of SPHK1 abrogated decreases in Th2 cytokines by ASC‐sEVs. Hence, S1P‐mediated decreases in Th2 cytokine production should be a mechanism to alleviate inflammation in an AD mouse model (Shin et al. 2020).

Importantly, we demonstrated that keratinocyte differentiation is decreased in SPHK1 inhibition, and ASC‐sEVs attenuate these decreases in keratinocyte differentiation (Figure 6). These results suggest that ASC‐sEVs are useful to normalize keratinocyte differentiation in not only AD but also with other skin conditions associated with abnormal differentiation, such as psoriasis (Cheng et al. 2018) and systemic sclerosis (Lee, Jeon, et al. 2024; Lee, Won, et al. 2024).

S1P has pro‐mitogenic properties (Uchida and Park 2021), while S1P promotes keratinocyte differentiation (Schuppel et al. 2008). Thus, S1P can normalize differentiation either directly or indirectly via suppression of Th2 cytokine production.

Because S1P promotes immune cell migration by S1P receptor activation that then aggravates skin inflammation, the inhibition of the S1P receptor is useful to alleviate this inflammation (Kleuser and Baumer 2023; Masuda‐Kuroki et al. 2024). However, our study suggests that local S1P deficiency is implicated in skin inflammation and abnormal differentiation, which inhibits competent epidermal permeability barrier formation. Therefore, S1P looks to play diverse roles in inflammatory responses.

LPA promotes skin inflammation and induces pruritus (Kittaka et al. 2017), while it also promotes keratinocyte differentiation (Sumitomo et al. 2019). Since LPA shows anti‐inflammatory activities by activation of ERK 1/2, serine/threonine phosphatases, and PI3 kinase signalling pathways (Fan et al. 2008), the possibility of a role of LPA and PA in suppressing skin inflammation in skin treated with ASC‐sEVs has not been excluded. However, PA levels in ASC‐sEVs are low. Thus, PA is probably not relevant to the alleviation of inflammation.

LXR activation suppresses inflammatory response and improves epidermal permeability barrier function in AD‐model mice (Hatano et al. 2010). Thus, although neither cholesterol, 25‐hydroxy sterol, nor 27‐hydroxysterol levels are enriched in ASC‐sEVs, there is a possibility that 25‐hydroxy sterol and 27‐hydroxysterol contribute to alleviating inflammation in AD treated with ASC‐sEVs. Nevertheless, our prior and current studies demonstrate that induction of S1P production can ameliorate Th2‐type skin inflammation.

In addition to global immune suppressors such as steroids, cyclosporin and tacrolimus, mechanism‐based anti‐inflammatory biologics, including IL‐13, IL‐31 antibodies, small molecules, JAK kinase inhibitors, PDE inhibitors and aryl hydrocarbon receptor (Ahr) modulators, have all have been developed to treat AD. The application of ASC‐sEVs could be an alternative therapeutic strategy for ameliorating AD and other inflammatory diseases.

## Author Contributions

**Kyong‐Oh Shin**: conceptualization (equal), data curation (lead), formal analysis (lead), investigation (lead), methodology (lead), validation (lead), writing – original draft (supporting), writing – review and editing (supporting). **Jun Ho Lee**: formal analysis, investigation, writing – original draft, writing – review and editing. **Seungwoo Chae**: investigation (supporting). **Karin Goto**: investigation (supporting). **Hahyun An**: investigation (supporting). **Joan S. Wakefield**: writing – review and editing (equal). **Dae Hyun Ha**: formal analysis (equal). **Healim Lee**: formal analysis (equal). **Kyojin Lee**: formal analysis (equal). **Hyunju Lee**: formal analysis (equal). **Ella Shin**: formal analysis (equal). **Min Ji Kang**: investigation. **Sinhee Lee**: investigation (supporting). **Yoshikazu Uchida**: conceptualization (equal), data curation (supporting), formal analysis (supporting), validation (supporting), visualization (equal), writing – original draft (lead), writing – review and editing (lead). **Byong Seung Cho**: conceptualization (supporting), investigation (supporting). **Kyungho Park**: conceptualization (lead), data curation (equal), formal analysis (equal), funding acquisition (lead), investigation (equal), methodology (supporting), project administration (lead), resources (lead), supervision (lead), validation (equal), visualization (equal), writing – original draft (equal), writing – review and editing (equal).

## Conflicts of Interest

B.S.C., J.H.L., D.H.H., H.L., K.L., H.L., E.S. and M.J.K. are current employees of ExoCoBio Inc. B.S.C. is the CEO of ExoCoBio Inc. B.S.C., J.H.L. and D.H.H. are shareholders of ExoCoBio Inc. KOS holds stock in LaSS.

## Supporting information



Supplemental Tables 1–15

## Data Availability

The data that support the findings of this study are available from the corresponding authors upon reasonable request.
