# Supplementary material for: Small EVs From Adipose‐Derived MSCs Modulate Epidermal Barrier and Inflammation Via Sphingosine‐1‐Phosphate Signaling Pathway
Source: J Extracell Vesicles. 2025 Jul 22;14(7):e70121. doi: 10.1002/jev2.70121 (PMC12281460; doi:10.1002/jev2.70121)
Supplement: Supplementary file 1 — Supplemental Tables 1–15 [file JEV2-14-e70121-s001.docx]

Supplementary Information for

**A proposed pathway for small extracellular vesicles derived from human adipose tissue-derived mesenchymal stem cells (ASC-sEVs) to ameliorate atopic dermatitis-like skin inflammation through characterization of lipid and ceramide metabolic enzyme profiles**

Kyong-Oh Shin^1,2^, Jun Ho Lee^3^, Seungwoo Chae^1^, Karin Goto^1^, Hahyun An^1^, Joan S. Wakefield^4^, Dae Hyun Ha^3^, Healim Lee^3^, Kyojin Lee^3^, Hyunju Lee^3^, Ella Shin^3^, Min Ji Kang^3^, Sinhee Lee^5^, Yoshikazu Uchida^1*^, Byong Seung Cho^3^, Kyungho Park^1*^

^1^Department of Food Science and Nutrition, Convergence Program of Material Science for Medicine and Pharmaceutics, Hallym University, Chuncheon, Republic of Korea, ^2^LaSS Lipid Institute (LLI), LaSS Inc., Chuncheon, Republic of Korea; ^3^ExoCoBio Exosome Institute (EEI), ExoCoBio Inc., STE 306, 19 Gasan digital 1-ro, Geumcheon-gu, Seoul, Republic of Korea, ^4^Department of Dermatology, School of Medicine, University of California San Francisco, Department of Veterans Affairs Medical Center, San Francisco, Northern California Institute for Research and Education, San Francisco, California, USA, ^5^Department of Cosmetic Industry, Chungbuk National University, Cheongju, Republic of Korea

* Correspondence: [Yoshikazu.Uchida@ucsf.edu](mailto:Yoshikazu.Uchida@ucsf.edu) and [Kyungho.Park@hallym.ac.kr](mailto:Kyungho.Park@hallym.ac.kr)

**This file includes:**

- SUPPLEMENTARY Tables

Supplementary Table 1

Supplementary Table 2

Supplementary Table 3

Supplementary Table 4

Supplementary Table 5

Supplementary Table 6

Supplementary Table 7

Supplementary Table 8

Supplementary Table 9

Supplementary Table 10

Supplementary Table 11

Supplementary Table 12

Supplementary Table 13

Supplementary Table 14

Supplementary Table 15

**Supplemental Table 1. Characterization of ASC Cell Bank**

| ASC  cell line | Donor information | | | Adventitious virus screening test | | | Sterility test | | | MSC-specific cell surface marker expression level  (Relative expression, %) | | | MSC-negative cell surface marker expression level  (Relative expression, %) | | | Differential potency | | |
| --- | --- | --- | --- | --- | --- | --- | --- | --- | --- | --- | --- | --- | --- | --- | --- | --- | --- | --- |
|  | Sex | Age (yr) | BMI | HBV | HCV | HIV | Endo toxin  (EU/ml) | Steri-lity | Myco plasma | CD29 | CD90 | CD105 | CD31 | CD45 | HLA-DR | Adipocytes | Osteoblasts | Chondrocytes |
| P003  (Donor A) | F | 25 | 25.1 | - | - | - | < 0.5 | - | - | ≥ 80 | ≥ 80 | ≥ 80 | < 3 | < 3 | < 3 | + | + | + |
| P004  (Donor B) | F | 22 | 26.1 | - | - | - | < 0.5 | - | - | ≥ 80 | ≥ 80 | ≥ 80 | < 3 | < 3 | < 3 | + | + | + |
| P006  (Donor C) | F | 29 | 26.74 | - | - | - | < 0.5 | - | - | ≥ 80 | ≥ 80 | ≥ 80 | < 3 | < 3 | < 3 | + | + | + |
| P008  (Donor D) | F | 28 | 22.27 | - | - | - | < 0.5 | - | - | ≥ 80 | ≥ 80 | ≥ 80 | < 3 | < 3 | < 3 | + | + | + |

The four donor cell screening result is confirmed properly for adventitious virus (HBV, HCV, HIV). The sterility test (endotoxin, sterility test, mycoplasma test) of four donor cells confirmed that the result was passed. The MSC-specific surface marker expression test of three donor cells confirmed high expression levels of CD29, CD73, and CD105 and very low levels of CD31. The value is the median percentage expression of MSC-specific surface markers. Three donor cells' differential potency (adipocyte, osteoblast, chondrocyte) confirmed that all are suitable. ASC, adipose tissue-derived mesenchymal stromal cells; -, negative; +, positive.

**Supplemental Table 2. Lipid content in small extracellular vesicles (sEVs) originating from human adipose-derived mesenchymal stem cells and their donor cells (pmol/mg protein)**

|  | ASC-sEVs | | | | | | Donor Cells | | | | | |  |
| --- | --- | --- | --- | --- | --- | --- | --- | --- | --- | --- | --- | --- | --- |
|  | Donor A | Donor B | Donor C | Donor D | Mean | SD | Donor A | Donor B | Donor C | Donor D | Mean | SD | t-test |
| Cholesterol | 41.65 | 41.15 | 31.38 | 69.99 | 46.04 | 16.6503 | 51.34 | 45.76 | 35.15 | 77.06 | 52.33 | 17.80 | 0.6245 |
| Cholesterol ester | 5.26 | 5.21 | 4.41 | 5.32 | 5.05 | 0.42903 | 221.35 | 219.06 | 305.01 | 364.46 | 277.47 | 70.44 | 0.0002 |
| Other sterols* | 10.73 | 6.71 | 7.44 | 8.99 | 8.47 | 1.78293 | 21.82 | 8.5 | 14.78 | 8 | 13.28 | 6.48 | 0.2024 |
| FFA | 2876.48 | 3309.68 | 2171.47 | 2769.54 | 2781.79 | 469.139 | 999.79 | 1946.39 | 1280.04 | 2215.6 | 1610.46 | 566.02 | 0.0189 |
| DG | 130.65 | 457.63 | 324.83 | 549.57 | 365.67 | 181.822 | 131.44 | 415.53 | 299.98 | 495.52 | 335.62 | 158.02 | 0.8113 |
| TG | 785.07 | 1203.05 | 703.09 | 1320.24 | 1002.86 | 304.467 | 606.3 | 1053.09 | 618.15 | 1152.02 | 857.39 | 286.00 | 0.5122 |
| NS | 1058.04 | 1461.34 | 1052.91 | 1777.66 | 1337.49 | 350.317 | 370.98 | 860.79 | 621.68 | 1045.97 | 724.86 | 292.95 | 0.0364 |
| NDS | 256 | 354.52 | 212.4 | 296.71 | 279.91 | 60.4929 | 88.66 | 208.13 | 124.93 | 174.28 | 149.00 | 52.78 | 0.0172 |
| Sphingoid base | 70.21 | 97.46 | 58.35 | 118.45 | 86.12 | 27.0686 | 64.02 | 91.4 | 56.12 | 110.33 | 80.47 | 25.00 | 0.7694 |
| SM | 2773.16 | 4593.85 | 3912.79 | 5218 | 4124.45 | 1046.74 | 1313.29 | 3154.95 | 2688.15 | 3582.86 | 2684.81 | 984.65 | 0.0920 |
| Lyso SM | 10.96 | 18.48 | 15.43 | 20.66 | 16.38 | 4.20346 | 41.9 | 240.59 | 209.29 | 263 | 188.70 | 100.31 | 0.0139 |
| PA | 200.1 | 278.45 | 165.96 | 336.43 | 245.24 | 76.9014 | 755.98 | 1026.23 | 638.84 | 1223.37 | 911.11 | 263.92 | 0.0029 |
| PC | 22914.2 | 17569.1 | 13503.2 | 24060.1 | 19511.69 | 4903.77 | 17376.3 | 15302.5 | 11765.2 | 20949.8 | 16348.44 | 3843.99 | 0.3491 |
| PE | 15234.4 | 17241.5 | 11000.6 | 17915.9 | 15348.13 | 3114.06 | 11563.5 | 15017.5 | 9587.94 | 15604.2 | 12943.30 | 2860.38 | 0.2987 |
| PS | 1289.17 | 1781.81 | 962.87 | 1805.1 | 1459.74 | 407.83 | 1008.32 | 1567.58 | 855.09 | 1587.87 | 1254.72 | 378.28 | 0.4888 |
| Total | 47656.1 | 48420 | 34127.2 | 56272.7 |  |  | 34615 | 41158 | 29100.3 | 48854.3 |  |  |  |

ASC-sEVs, small extracellular vesicles from human adipose tissue-derived mesenchymal stem cells; FFA, free fatty acid; DG, diglyceride; TG, triglyceride; NS, ceramide NS; NDS, ceramide NDS; SM, sphingomyelin; PA, phosphatidic acid; PC, phosphatidylcholine; PE, phosphatidylethanoamine; PS, phosphatidylserine.

**Supplemental Table 3. Free fatty acids content in small extracellular vesicles (sEVs) originating from human adipose-derived mesenchymal stem cells and their donor cells (pmol/mg protein)**

|  | ASC-sEVs | | | | | | | | Donor Cells | | | | | | | |
| --- | --- | --- | --- | --- | --- | --- | --- | --- | --- | --- | --- | --- | --- | --- | --- | --- |
|  | Donor A | | Donor B | | Donor C | | Donor D | | Donor A | | Donor B | | Donor C | | Donor D | |
|  | Mean | SD | Mean | SD | Mean | SD | Mean | SD | Mean | SD | Mean | SD | Mean | SD | Mean | SD |
| C14 | 17.38 | 1.62 | 20.11 | 1.86 | 13.05 | 1.22 | 22.79 | 2.12 | 3.83 | 0.24 | 6.35 | 0.47 | 4.28 | 0.31 | 7.08 | 0.53 |
| C16 | 765.13 | 32.55 | 880.02 | 37.43 | 577.83 | 24.59 | 1002.64 | 42.65 | 1.67 | 0.06 | 2.11 | 0.12 | 1.50 | 0.08 | 2.26 | 0.14 |
| C16:1 | 563.95 | 48.84 | 648.65 | 56.17 | 425.87 | 36.89 | 739.01 | 64.00 | 2.70 | 0.22 | 4.14 | 0.42 | 2.83 | 0.28 | 4.57 | 0.48 |
| C18 | 719.74 | 52.18 | 827.81 | 60.01 | 543.54 | 39.41 | 943.15 | 68.37 | 2.17 | 0.16 | 3.10 | 0.32 | 2.15 | 0.21 | 3.38 | 0.36 |
| C18:1 | 232.75 | 16.45 | 267.78 | 18.92 | 175.72 | 12.43 | 305.01 | 21.56 | 20.75 | 1.13 | 39.59 | 2.22 | 26.11 | 1.46 | 44.96 | 2.53 |
| C18:2 | 337.26 | 10.57 | 387.96 | 12.16 | 254.65 | 7.98 | 441.95 | 13.85 | 7.19 | 0.30 | 12.95 | 0.59 | 8.62 | 0.39 | 14.60 | 0.67 |
| C18:3 | 79.07 | 2.71 | 91.04 | 3.12 | 59.65 | 2.05 | 103.62 | 3.55 | 13.57 | 0.52 | 25.49 | 1.02 | 16.85 | 0.67 | 28.89 | 1.17 |
| C20 | 8.46 | 0.69 | 9.84 | 0.79 | 6.31 | 0.52 | 11.09 | 0.91 | 10.80 | 0.49 | 20.03 | 0.96 | 13.27 | 0.63 | 22.68 | 1.09 |
| C20:1 | 2.17 | 0.18 | 2.61 | 0.21 | 1.56 | 0.14 | 2.86 | 0.24 | 555.81 | 19.09 | 1084.77 | 37.49 | 713.14 | 24.63 | 1235.17 | 42.72 |
| C20:4 | 5.18 | 0.63 | 6.07 | 0.72 | 3.84 | 0.47 | 6.80 | 0.82 | 294.57 | 10.68 | 572.78 | 20.97 | 376.75 | 13.77 | 651.92 | 23.90 |
| C20:5 | 3.63 | 0.47 | 4.29 | 0.54 | 2.67 | 0.36 | 4.77 | 0.62 | 149.42 | 4.27 | 288.83 | 8.39 | 190.15 | 5.51 | 328.51 | 9.56 |
| C22 | 57.83 | 3.29 | 66.62 | 3.79 | 43.61 | 2.49 | 75.80 | 4.32 | 999.79 | 12.66 | 1946.39 | 24.87 | 1280.04 | 16.33 | 2215.60 | 28.34 |
| C22:1 | 18.26 | 0.88 | 21.11 | 1.01 | 13.72 | 0.66 | 23.94 | 1.15 | 10.76 | 0.95 | 7.82 | 0.64 | 5.40 | 0.42 | 8.74 | 0.73 |
| C24 | 36.89 | 1.52 | 42.54 | 1.75 | 27.79 | 1.15 | 48.35 | 1.99 | 448.52 | 19.06 | 302.54 | 12.83 | 198.97 | 8.43 | 344.57 | 14.62 |
| C24:1 | 28.79 | 1.42 | 33.22 | 1.64 | 21.67 | 1.08 | 37.74 | 1.87 | 330.74 | 28.59 | 223.24 | 19.25 | 146.89 | 12.64 | 254.21 | 21.94 |
| Saturated FFA | 1605.43 | 55.69 | 1846.93 | 64.05 | 1212.13 | 42.06 | 2103.83 | 72.98 | 421.94 | 30.55 | 284.65 | 20.57 | 187.22 | 13.51 | 324.18 | 23.43 |
| Mono saturated FFA | 845.92 | 31.15 | 973.38 | 35.82 | 638.55 | 23.53 | 1108.56 | 40.82 | 136.85 | 9.63 | 92.71 | 6.48 | 61.15 | 4.26 | 105.47 | 7.39 |
| Poly saturated FFA | 425.13 | 12.46 | 489.36 | 14.33 | 320.80 | 9.41 | 557.15 | 16.33 | 198.03 | 6.19 | 133.90 | 4.17 | 88.21 | 2.74 | 152.40 | 4.75 |

ASC-sEVs, small extracellular vesicles from human adipose tissue-derived mesenchymal stem cells; FFA, free fatty acid

**Supplemental Table 4. Diglyceride content in small extracellular vesicles (sEVs) originating from human adipose-derived mesenchymal stem cells and their donor cells (pmol/mg protein)**

|  | ASC-sEVs | | | | | | | | Donor Cells | | | | | | | |
| --- | --- | --- | --- | --- | --- | --- | --- | --- | --- | --- | --- | --- | --- | --- | --- | --- |
|  | Donor A | | Donor B | | Donor C | | Donor D | | Donor A | | Donor B | | Donor C | | Donor D | |
|  | Mean | SD | Mean | SD | Mean | SD | Mean | SD | Mean | SD | Mean | SD | Mean | SD | Mean | SD |
| C14 | 0.25 | 0.01 | 1.21 | 0.03 | 0.60 | 0.02 | 1.09 | 0.04 | 1.82 | 0.01 | 1.92 | 0.03 | 1.39 | 0.02 | 1.82 | 0.03 |
| C16 | 21.74 | 1.05 | 75.33 | 3.63 | 54.10 | 2.62 | 91.33 | 4.41 | 18.08 | 0.80 | 66.41 | 3.15 | 47.93 | 2.28 | 80.33 | 3.84 |
| C18 | 4.34 | 0.26 | 15.30 | 0.91 | 10.77 | 0.66 | 18.25 | 1.11 | 4.91 | 0.20 | 14.18 | 0.79 | 10.24 | 0.57 | 16.75 | 0.96 |
| 14:0-16:0 | 2.59 | 0.20 | 9.30 | 0.70 | 6.44 | 0.51 | 10.94 | 0.86 | 3.59 | 0.15 | 8.96 | 0.61 | 6.47 | 0.44 | 10.39 | 0.74 |
| 14:0-18:0 | 4.37 | 0.40 | 15.44 | 1.38 | 10.87 | 1.00 | 18.42 | 1.68 | 4.94 | 0.30 | 14.30 | 1.20 | 10.33 | 0.87 | 16.89 | 1.46 |
| 16:0-14:0 | 27.63 | 0.92 | 95.67 | 3.17 | 68.77 | 2.29 | 116.09 | 3.85 | 22.54 | 0.69 | 84.10 | 2.75 | 60.70 | 1.99 | 101.8 | 3.35 |
| 16:0-18:0 | 10.31 | 1.00 | 35.91 | 3.46 | 25.64 | 2.50 | 43.34 | 4.21 | 9.43 | 0.76 | 32.11 | 3.01 | 23.18 | 2.17 | 38.57 | 3.67 |
| 16:0-18:1 | 0.84 | 0.08 | 3.25 | 0.27 | 2.07 | 0.20 | 3.58 | 0.33 | 2.26 | 0.06 | 3.70 | 0.24 | 2.67 | 0.17 | 3.98 | 0.29 |
| 16:0-18:2 | 5.30 | 0.45 | 18.64 | 1.56 | 13.18 | 1.13 | 22.31 | 1.90 | 5.64 | 0.34 | 17.09 | 1.36 | 12.33 | 0.98 | 20.28 | 1.65 |
| 16:0-20:4 | 0.16 | 0.02 | 0.90 | 0.06 | 0.38 | 0.05 | 0.72 | 0.08 | 1.75 | 0.01 | 1.65 | 0.06 | 1.20 | 0.04 | 1.50 | 0.07 |
| 16:0-22:6 | 0.71 | 0.05 | 2.79 | 0.18 | 1.74 | 0.13 | 3.02 | 0.22 | 2.16 | 0.04 | 3.30 | 0.16 | 2.39 | 0.11 | 3.50 | 0.19 |
| 18:0-14:0 | 5.90 | 0.63 | 20.69 | 2.18 | 14.66 | 1.58 | 24.81 | 2.66 | 6.09 | 0.48 | 18.87 | 1.90 | 13.62 | 1.37 | 22.46 | 2.31 |
| 18:0-16:0 | 1.29 | 0.07 | 4.78 | 0.24 | 3.18 | 0.17 | 5.44 | 0.29 | 2.60 | 0.05 | 5.03 | 0.21 | 3.63 | 0.15 | 5.61 | 0.25 |
| 18:0-18:1 | 7.07 | 0.68 | 24.73 | 2.35 | 17.57 | 1.70 | 29.73 | 2.86 | 6.98 | 0.52 | 22.38 | 2.04 | 16.16 | 1.48 | 26.73 | 2.49 |
| 18:0-18:2 | 14.71 | 0.44 | 51.09 | 1.54 | 36.60 | 1.11 | 61.82 | 1.87 | 12.76 | 0.34 | 45.32 | 1.34 | 32.71 | 0.96 | 54.65 | 1.63 |
| 18:0-20:4 | 11.75 | 0.56 | 40.89 | 1.92 | 29.24 | 1.39 | 49.40 | 2.34 | 10.52 | 0.42 | 36.44 | 1.67 | 26.31 | 1.21 | 43.85 | 2.04 |
| 18:0-22:6 | 0.32 | 0.02 | 1.43 | 0.05 | 0.76 | 0.04 | 1.37 | 0.06 | 1.87 | 0.01 | 2.12 | 0.05 | 1.53 | 0.03 | 2.06 | 0.06 |
| 18:1-14:0 | 0.79 | 0.06 | 3.07 | 0.21 | 1.94 | 0.15 | 3.36 | 0.26 | 2.23 | 0.05 | 3.54 | 0.19 | 2.56 | 0.13 | 3.80 | 0.23 |
| 18:1-16:0 | 3.13 | 0.20 | 11.15 | 0.68 | 7.78 | 0.49 | 13.20 | 0.83 | 4.00 | 0.15 | 10.57 | 0.59 | 7.63 | 0.43 | 12.35 | 0.72 |
| 18:1-18:0 | 7.45 | 0.66 | 26.06 | 2.28 | 18.54 | 1.65 | 31.35 | 2.78 | 7.27 | 0.50 | 23.54 | 1.98 | 17.00 | 1.43 | 28.14 | 2.41 |

ASC-sEVs, small extracellular vesicles from human adipose tissue-derived mesenchymal stem cells

**Supplemental Table 5. TG content in small extracellular vesicles (sEVs) originating from human adipose-derived mesenchymal stem cells and their donor cells (pmol/mg protein)**

|  | ASC-sEVs | | | | | | | | Donor Cells | | | | | | | |
| --- | --- | --- | --- | --- | --- | --- | --- | --- | --- | --- | --- | --- | --- | --- | --- | --- |
|  | Donor A | | Donor B | | Donor C | | Donor D | | Donor A | | Donor B | | Donor C | | Donor D | |
|  | Mean | SD | Mean | SD | Mean | SD | Mean | SD | Mean | SD | Mean | SD | Mean | SD | Mean | SD |
| TG(40:0) | 0.21 | 0.02 | 0.47 | 0.02 | 0.18 | 0.01 | 0.37 | 0.03 | 0.32 | 0.01 | 0.50 | 0.02 | 0.25 | 0.01 | 0.41 | 0.02 |
| TG(40:1) | 0.31 | 0.02 | 0.62 | 0.03 | 0.27 | 0.02 | 0.53 | 0.04 | 0.40 | 0.02 | 0.63 | 0.03 | 0.32 | 0.02 | 0.55 | 0.03 |
| TG(42:0) | 0.33 | 0.02 | 0.66 | 0.03 | 0.29 | 0.02 | 0.58 | 0.03 | 0.42 | 0.01 | 0.66 | 0.02 | 0.34 | 0.01 | 0.59 | 0.03 |
| TG(42:1) | 0.05 | 0.00 | 0.23 | 0.01 | 0.04 | 0.00 | 0.10 | 0.01 | 0.20 | 0.00 | 0.29 | 0.00 | 0.12 | 0.00 | 0.18 | 0.01 |
| TG(42:2) | 0.41 | 0.02 | 0.77 | 0.03 | 0.36 | 0.02 | 0.70 | 0.04 | 0.47 | 0.02 | 0.76 | 0.03 | 0.40 | 0.02 | 0.70 | 0.03 |
| TG(42:3) | 0.11 | 0.01 | 0.33 | 0.01 | 0.09 | 0.01 | 0.21 | 0.01 | 0.25 | 0.00 | 0.37 | 0.01 | 0.17 | 0.00 | 0.27 | 0.01 |
| TG(44:0) | 0.65 | 0.02 | 1.14 | 0.02 | 0.57 | 0.01 | 1.11 | 0.03 | 0.65 | 0.01 | 1.08 | 0.02 | 0.59 | 0.01 | 1.05 | 0.02 |
| TG(44:1) | 2.14 | 0.24 | 3.40 | 0.36 | 1.91 | 0.21 | 3.62 | 0.40 | 1.78 | 0.18 | 3.05 | 0.31 | 1.75 | 0.18 | 3.23 | 0.34 |
| TG(44:2) | 1.69 | 0.15 | 2.71 | 0.23 | 1.50 | 0.13 | 2.85 | 0.25 | 1.44 | 0.11 | 2.45 | 0.20 | 1.39 | 0.12 | 2.57 | 0.22 |
| TG(46:0) | 1.36 | 0.08 | 2.22 | 0.12 | 1.21 | 0.07 | 2.31 | 0.13 | 1.19 | 0.06 | 2.02 | 0.10 | 1.14 | 0.06 | 2.09 | 0.11 |
| TG(46:1) | 9.87 | 0.82 | 15.13 | 1.24 | 8.84 | 0.73 | 16.59 | 1.37 | 7.63 | 0.62 | 13.25 | 1.08 | 7.77 | 0.64 | 14.52 | 1.19 |
| TG(46:2) | 21.89 | 2.04 | 33.37 | 3.09 | 19.61 | 1.83 | 36.78 | 3.42 | 16.73 | 1.54 | 29.12 | 2.69 | 17.15 | 1.59 | 32.09 | 2.98 |
| TG(46:3) | 6.57 | 1.00 | 10.13 | 1.52 | 5.88 | 0.90 | 11.06 | 1.68 | 5.14 | 0.76 | 8.90 | 1.32 | 5.21 | 0.78 | 9.71 | 1.46 |
| TG(46:4) | 1.38 | 0.06 | 2.25 | 0.09 | 1.23 | 0.06 | 2.34 | 0.10 | 1.21 | 0.05 | 2.05 | 0.08 | 1.16 | 0.05 | 2.12 | 0.09 |
| TG(46:5) | 0.24 | 0.01 | 0.52 | 0.01 | 0.21 | 0.01 | 0.42 | 0.01 | 0.35 | 0.01 | 0.54 | 0.01 | 0.27 | 0.01 | 0.46 | 0.01 |
| TG(48:0) | 0.90 | 0.11 | 1.53 | 0.17 | 0.80 | 0.10 | 1.54 | 0.19 | 0.85 | 0.08 | 1.41 | 0.15 | 0.78 | 0.09 | 1.42 | 0.16 |
| TG(48:1) | 37.64 | 2.30 | 57.29 | 3.48 | 33.73 | 2.06 | 63.26 | 3.86 | 28.66 | 1.74 | 49.93 | 3.03 | 29.44 | 1.79 | 55.12 | 3.36 |
| TG(48:2) | 59.97 | 2.52 | 91.19 | 3.83 | 53.75 | 2.26 | 100.77 | 4.24 | 45.55 | 1.91 | 79.42 | 3.33 | 46.85 | 1.97 | 87.75 | 3.69 |
| TG(48:3) | 45.35 | 4.97 | 69.00 | 7.55 | 40.65 | 4.46 | 76.21 | 8.36 | 34.49 | 3.76 | 60.12 | 6.57 | 35.45 | 3.88 | 66.39 | 7.27 |
| TG(48:4) | 15.23 | 1.19 | 23.28 | 1.80 | 13.65 | 1.06 | 25.61 | 1.99 | 11.69 | 0.90 | 20.34 | 1.57 | 11.96 | 0.93 | 22.37 | 1.73 |
| TG(48:5) | 2.43 | 0.11 | 3.83 | 0.16 | 2.17 | 0.10 | 4.09 | 0.18 | 2.00 | 0.08 | 3.42 | 0.14 | 1.97 | 0.08 | 3.65 | 0.16 |
| TG(49:1) | 4.72 | 0.41 | 7.32 | 0.62 | 4.22 | 0.37 | 7.95 | 0.69 | 3.74 | 0.31 | 6.45 | 0.54 | 3.76 | 0.32 | 7.00 | 0.60 |
| TG(49:2) | 4.72 | 0.36 | 7.32 | 0.55 | 4.22 | 0.33 | 7.95 | 0.61 | 3.74 | 0.28 | 6.46 | 0.48 | 3.76 | 0.28 | 7.00 | 0.53 |
| TG(49:3) | 4.44 | 0.33 | 6.90 | 0.50 | 3.97 | 0.30 | 7.48 | 0.55 | 3.53 | 0.25 | 6.09 | 0.43 | 3.54 | 0.26 | 6.59 | 0.48 |
| TG(50:0) | 0.21 | 0.01 | 0.47 | 0.01 | 0.18 | 0.01 | 0.37 | 0.02 | 0.32 | 0.01 | 0.49 | 0.01 | 0.24 | 0.01 | 0.41 | 0.01 |
| TG(50:1) | 30.14 | 2.08 | 45.90 | 3.16 | 27.01 | 1.86 | 50.65 | 3.49 | 22.97 | 1.57 | 40.02 | 2.75 | 23.58 | 1.62 | 44.15 | 3.04 |
| TG(50:2) | 107.93 | 5.72 | 163.99 | 8.68 | 96.74 | 5.12 | 181.34 | 9.60 | 81.85 | 4.33 | 142.76 | 7.55 | 84.25 | 4.46 | 157.85 | 8.36 |
| TG(50:3) | 112.29 | 5.99 | 170.61 | 9.09 | 100.65 | 5.37 | 188.66 | 10.06 | 85.15 | 4.53 | 148.51 | 7.91 | 87.65 | 4.67 | 164.22 | 8.75 |
| TG(50:4) | 56.52 | 1.32 | 85.94 | 2.00 | 50.65 | 1.18 | 94.96 | 2.21 | 42.94 | 1.00 | 74.86 | 1.74 | 44.15 | 1.03 | 82.71 | 1.93 |
| TG(50:5) | 17.04 | 0.34 | 26.02 | 0.51 | 15.27 | 0.30 | 28.65 | 0.57 | 13.06 | 0.26 | 22.73 | 0.45 | 13.37 | 0.26 | 25.01 | 0.49 |
| TG(51:1) | 0.35 | 0.03 | 0.69 | 0.05 | 0.31 | 0.03 | 0.61 | 0.05 | 0.43 | 0.02 | 0.69 | 0.04 | 0.36 | 0.02 | 0.62 | 0.05 |
| TG(51:2) | 3.01 | 0.17 | 4.73 | 0.26 | 2.69 | 0.15 | 5.08 | 0.29 | 2.44 | 0.13 | 4.20 | 0.23 | 2.43 | 0.13 | 4.51 | 0.25 |
| TG(51:3) | 6.44 | 0.53 | 9.93 | 0.81 | 5.76 | 0.48 | 10.84 | 0.90 | 5.04 | 0.40 | 8.72 | 0.70 | 5.10 | 0.42 | 9.51 | 0.78 |
| TG(51:4) | 4.91 | 0.46 | 7.60 | 0.69 | 4.39 | 0.41 | 8.26 | 0.77 | 3.88 | 0.35 | 6.70 | 0.60 | 3.91 | 0.36 | 7.27 | 0.67 |
| TG(51:5) | 2.10 | 0.07 | 3.33 | 0.11 | 1.87 | 0.06 | 3.54 | 0.12 | 1.75 | 0.05 | 2.99 | 0.09 | 1.71 | 0.06 | 3.17 | 0.11 |
| TG(52:0) | 0.20 | 0.01 | 0.46 | 0.01 | 0.17 | 0.01 | 0.36 | 0.02 | 0.32 | 0.01 | 0.49 | 0.01 | 0.24 | 0.01 | 0.40 | 0.01 |
| TG(52:1) | 5.62 | 0.20 | 8.69 | 0.31 | 5.03 | 0.18 | 9.47 | 0.34 | 4.42 | 0.16 | 7.65 | 0.27 | 4.47 | 0.16 | 8.32 | 0.30 |
| TG(52:2) | 19.29 | 2.39 | 29.43 | 3.63 | 17.28 | 2.14 | 32.42 | 4.01 | 14.76 | 1.81 | 25.69 | 3.16 | 15.12 | 1.86 | 28.29 | 3.49 |
| TG(52:3) | 51.93 | 3.17 | 78.99 | 4.81 | 46.54 | 2.84 | 87.26 | 5.32 | 39.47 | 2.40 | 68.80 | 4.18 | 40.58 | 2.47 | 76.01 | 4.63 |
| TG(52:4) | 52.05 | 2.19 | 79.16 | 3.33 | 46.65 | 1.96 | 87.46 | 3.68 | 39.56 | 1.66 | 68.96 | 2.89 | 40.67 | 1.71 | 76.17 | 3.20 |
| TG(52:5) | 44.38 | 4.87 | 67.51 | 7.39 | 39.77 | 4.36 | 74.57 | 8.18 | 33.75 | 3.68 | 58.82 | 6.43 | 34.69 | 3.80 | 64.96 | 7.11 |
| TG(53:2) | 0.37 | 0.03 | 0.71 | 0.04 | 0.32 | 0.03 | 0.63 | 0.05 | 0.44 | 0.02 | 0.70 | 0.04 | 0.37 | 0.02 | 0.64 | 0.04 |
| TG(53:3) | 1.44 | 0.06 | 2.34 | 0.10 | 1.28 | 0.06 | 2.44 | 0.11 | 1.25 | 0.05 | 2.12 | 0.08 | 1.20 | 0.05 | 2.21 | 0.09 |
| TG(53:4) | 1.54 | 0.15 | 2.50 | 0.23 | 1.38 | 0.13 | 2.61 | 0.25 | 1.33 | 0.11 | 2.26 | 0.20 | 1.28 | 0.12 | 2.36 | 0.22 |
| TG (53:5) | 1.85 | 0.05 | 2.96 | 0.07 | 1.65 | 0.04 | 3.12 | 0.08 | 1.56 | 0.04 | 2.66 | 0.06 | 1.52 | 0.04 | 2.80 | 0.07 |
| TG(54:0) | 0.78 | 0.04 | 1.34 | 0.05 | 0.69 | 0.03 | 1.34 | 0.06 | 0.76 | 0.03 | 1.26 | 0.05 | 0.69 | 0.03 | 1.25 | 0.05 |
| TG(54:1) | 0.83 | 0.03 | 1.41 | 0.04 | 0.73 | 0.02 | 1.41 | 0.04 | 0.79 | 0.02 | 1.31 | 0.03 | 0.73 | 0.02 | 1.31 | 0.04 |
| TG(54:2) | 1.65 | 0.11 | 2.65 | 0.16 | 1.47 | 0.10 | 2.78 | 0.18 | 1.41 | 0.08 | 2.39 | 0.14 | 1.36 | 0.08 | 2.51 | 0.16 |
| TG(54:3) | 3.72 | 0.32 | 5.80 | 0.49 | 3.33 | 0.29 | 6.27 | 0.54 | 2.98 | 0.24 | 5.13 | 0.42 | 2.98 | 0.25 | 5.54 | 0.47 |
| TG(54:4) | 6.47 | 0.25 | 9.97 | 0.38 | 5.79 | 0.23 | 10.88 | 0.43 | 5.06 | 0.19 | 8.76 | 0.33 | 5.12 | 0.20 | 9.56 | 0.37 |
| TG(54:5) | 2.26 | 0.17 | 3.58 | 0.25 | 2.02 | 0.15 | 3.82 | 0.28 | 1.87 | 0.13 | 3.21 | 0.22 | 1.84 | 0.13 | 3.41 | 0.24 |
| TG(54:6) | 6.66 | 0.42 | 10.26 | 0.64 | 5.96 | 0.38 | 11.20 | 0.71 | 5.20 | 0.32 | 9.01 | 0.56 | 5.27 | 0.33 | 9.84 | 0.62 |
| TG(54:7) | 6.22 | 0.60 | 9.60 | 0.91 | 5.57 | 0.54 | 10.47 | 1.01 | 4.87 | 0.45 | 8.44 | 0.79 | 4.93 | 0.47 | 9.20 | 0.87 |
| TG(56:0) | 0.14 | 0.00 | 0.36 | 0.01 | 0.11 | 0.00 | 0.25 | 0.01 | 0.27 | 0.00 | 0.40 | 0.00 | 0.19 | 0.00 | 0.30 | 0.01 |
| TG(56:1) | 1.01 | 0.05 | 1.68 | 0.07 | 0.89 | 0.04 | 1.71 | 0.08 | 0.93 | 0.04 | 1.55 | 0.06 | 0.87 | 0.04 | 1.57 | 0.07 |
| TG(56:2) | 0.78 | 0.08 | 1.34 | 0.12 | 0.69 | 0.07 | 1.33 | 0.13 | 0.76 | 0.06 | 1.25 | 0.10 | 0.69 | 0.06 | 1.25 | 0.11 |
| TG(56:3) | 0.28 | 0.02 | 0.58 | 0.03 | 0.24 | 0.02 | 0.49 | 0.03 | 0.38 | 0.02 | 0.59 | 0.03 | 0.30 | 0.02 | 0.51 | 0.03 |
| TG(56:4) | 0.10 | 0.01 | 0.31 | 0.01 | 0.08 | 0.01 | 0.19 | 0.01 | 0.24 | 0.01 | 0.36 | 0.01 | 0.16 | 0.01 | 0.25 | 0.01 |
| TG(56:5) | 0.11 | 0.01 | 0.32 | 0.01 | 0.09 | 0.01 | 0.20 | 0.01 | 0.24 | 0.01 | 0.36 | 0.01 | 0.16 | 0.01 | 0.26 | 0.01 |
| TG(57:1) | 0.39 | 0.02 | 0.74 | 0.03 | 0.34 | 0.02 | 0.67 | 0.03 | 0.46 | 0.01 | 0.73 | 0.02 | 0.38 | 0.01 | 0.67 | 0.03 |
| TG(57:2) | 0.74 | 0.05 | 1.28 | 0.08 | 0.66 | 0.05 | 1.26 | 0.09 | 0.72 | 0.04 | 1.20 | 0.07 | 0.66 | 0.04 | 1.18 | 0.07 |
| TG(58:0) | 0.10 | 0.01 | 0.31 | 0.01 | 0.08 | 0.00 | 0.19 | 0.01 | 0.24 | 0.00 | 0.35 | 0.01 | 0.16 | 0.00 | 0.25 | 0.01 |
| TG(58:1) | 1.80 | 0.10 | 2.89 | 0.15 | 1.61 | 0.09 | 3.05 | 0.16 | 1.53 | 0.07 | 2.60 | 0.13 | 1.49 | 0.07 | 2.74 | 0.14 |
| TG(58:2) | 3.12 | 0.07 | 4.89 | 0.11 | 2.79 | 0.07 | 5.26 | 0.12 | 2.53 | 0.06 | 4.34 | 0.10 | 2.51 | 0.06 | 4.66 | 0.11 |
| TG(58:3) | 1.34 | 0.15 | 2.18 | 0.22 | 1.19 | 0.13 | 2.26 | 0.25 | 1.17 | 0.11 | 1.98 | 0.19 | 1.12 | 0.11 | 2.05 | 0.21 |
| TG(59:2) | 1.43 | 0.13 | 2.32 | 0.19 | 1.27 | 0.11 | 2.42 | 0.21 | 1.24 | 0.10 | 2.10 | 0.17 | 1.19 | 0.10 | 2.19 | 0.18 |
| TG(59:3) | 0.90 | 0.05 | 1.52 | 0.08 | 0.80 | 0.05 | 1.53 | 0.09 | 0.84 | 0.04 | 1.41 | 0.07 | 0.78 | 0.04 | 1.42 | 0.08 |
| TG(60:1) | 0.81 | 0.07 | 1.38 | 0.10 | 0.72 | 0.06 | 1.38 | 0.11 | 0.78 | 0.05 | 1.29 | 0.09 | 0.71 | 0.05 | 1.29 | 0.10 |
| TG(60:2) | 0.02 | 0.00 | 0.19 | 0.00 | 0.01 | 0.00 | 0.05 | 0.00 | 0.18 | 0.00 | 0.25 | 0.00 | 0.10 | 0.00 | 0.13 | 0.00 |
| TG(60:4) | 0.55 | 0.02 | 0.99 | 0.02 | 0.48 | 0.01 | 0.94 | 0.03 | 0.58 | 0.01 | 0.95 | 0.02 | 0.51 | 0.01 | 0.91 | 0.02 |
| TG(62:1) | 0.08 | 0.00 | 0.28 | 0.01 | 0.07 | 0.00 | 0.16 | 0.01 | 0.23 | 0.00 | 0.33 | 0.00 | 0.14 | 0.00 | 0.22 | 0.01 |
| TG(62:2) | 0.20 | 0.01 | 0.46 | 0.01 | 0.17 | 0.01 | 0.36 | 0.01 | 0.32 | 0.01 | 0.49 | 0.01 | 0.24 | 0.01 | 0.40 | 0.01 |
| TG(62:3) | 0.18 | 0.02 | 0.42 | 0.02 | 0.15 | 0.01 | 0.32 | 0.03 | 0.30 | 0.01 | 0.46 | 0.02 | 0.22 | 0.01 | 0.36 | 0.02 |
| TG(62:4) | 0.18 | 0.01 | 0.43 | 0.02 | 0.15 | 0.01 | 0.32 | 0.02 | 0.30 | 0.01 | 0.46 | 0.01 | 0.22 | 0.01 | 0.37 | 0.02 |

ASC-sEVs, small extracellular vesicles from human adipose tissue-derived mesenchymal stem cells

**Supplemental Table 6. Phosphatidic choline content in small extracellular vesicles (sEVs) originating from human adipose-derived mesenchymal stem cells and their donor cells (pmol/mg protein)**

|  | ASC-sEVs | | | | | | | | Donor Cells | | | | | | | |
| --- | --- | --- | --- | --- | --- | --- | --- | --- | --- | --- | --- | --- | --- | --- | --- | --- |
|  | Donor A | | Donor B | | Donor C | | Donor D | | Donor A | | Donor B | | Donor C | | Donor D | |
|  | Mean | SD | Mean | SD | Mean | SD | Mean | SD | Mean | SD | Mean | SD | Mean | SD | Mean | SD |
| 16:0 | 499.71 | 11.47 | 383.19 | 8.80 | 294.47 | 6.76 | 524.70 | 12.05 | 379.86 | 8.68 | 334.24 | 7.65 | 257.06 | 5.88 | 457.36 | 10.48 |
| 18:0 | 682.63 | 42.05 | 523.43 | 32.24 | 402.27 | 24.78 | 716.77 | 44.15 | 518.31 | 31.83 | 456.25 | 28.05 | 350.84 | 21.56 | 624.46 | 38.41 |
| 20:0 | 389.45 | 25.99 | 298.66 | 19.92 | 229.50 | 15.31 | 408.94 | 27.29 | 296.40 | 19.67 | 260.70 | 17.33 | 200.53 | 13.32 | 356.65 | 23.74 |
| 14:0-16:0 | 447.27 | 35.52 | 342.98 | 27.23 | 263.57 | 20.93 | 469.64 | 37.30 | 340.16 | 26.88 | 299.26 | 23.69 | 230.17 | 18.21 | 409.46 | 32.45 |
| 14:0-18:0 | 850.87 | 49.04 | 652.41 | 37.60 | 501.41 | 28.90 | 893.43 | 51.49 | 645.65 | 37.12 | 568.47 | 32.71 | 437.10 | 25.14 | 778.15 | 44.80 |
| 16:0-14:0 | 120.47 | 12.45 | 92.44 | 9.55 | 70.99 | 7.34 | 126.51 | 13.07 | 92.81 | 9.42 | 81.29 | 8.31 | 62.63 | 6.38 | 110.93 | 11.37 |
| 16:0-18:0 | 3039.27 | 293.27 | 2330.19 | 224.84 | 1791.04 | 172.82 | 3191.25 | 307.93 | 2302.05 | 221.97 | 2028.13 | 195.61 | 1559.07 | 150.36 | 2777.25 | 267.90 |
| 16:0-18:1 | 1427.80 | 127.75 | 1094.72 | 97.94 | 841.40 | 75.28 | 1499.20 | 134.14 | 1082.33 | 96.69 | 953.28 | 85.21 | 732.89 | 65.50 | 1305.18 | 116.70 |
| 16:0-18:2 | 143.31 | 9.83 | 109.94 | 7.54 | 84.44 | 5.80 | 150.48 | 10.33 | 110.10 | 7.44 | 96.52 | 6.56 | 74.34 | 5.04 | 131.79 | 8.98 |
| 16:0-20:4 | 180.94 | 15.87 | 138.80 | 12.17 | 106.62 | 9.35 | 190.00 | 16.67 | 138.58 | 12.02 | 121.62 | 10.59 | 93.63 | 8.14 | 166.17 | 14.50 |
| 16:0-22:6 | 3625.52 | 172.47 | 2779.64 | 132.23 | 2136.51 | 101.64 | 3806.80 | 181.10 | 2745.78 | 130.54 | 2419.16 | 115.04 | 1859.63 | 88.42 | 3312.79 | 157.55 |
| 18:0-14:0 | 167.72 | 11.66 | 128.66 | 8.94 | 98.83 | 6.87 | 176.11 | 12.25 | 128.57 | 8.83 | 112.80 | 7.78 | 86.85 | 5.98 | 154.09 | 10.65 |
| 18:0-16:0 | 87.75 | 5.07 | 67.35 | 3.88 | 51.70 | 2.99 | 92.14 | 5.32 | 68.04 | 3.84 | 59.46 | 3.38 | 45.85 | 2.60 | 81.04 | 4.63 |
| 18:0-18:1 | 5635.68 | 306.93 | 4320.76 | 235.31 | 3321.10 | 180.87 | 5917.47 | 322.27 | 4267.27 | 232.31 | 3759.93 | 204.72 | 2890.23 | 157.36 | 5149.07 | 280.38 |
| 18:0-18:2 | 182.15 | 4.81 | 139.73 | 3.69 | 107.34 | 2.83 | 191.27 | 5.05 | 139.50 | 3.64 | 122.43 | 3.21 | 94.25 | 2.47 | 167.28 | 4.39 |
| 18:0-20:4 | 936.30 | 71.22 | 717.91 | 54.60 | 551.76 | 41.97 | 983.12 | 74.78 | 710.31 | 53.91 | 625.45 | 47.51 | 480.90 | 36.51 | 856.19 | 65.06 |
| 18:0-22:6 | 560.18 | 33.41 | 429.55 | 25.61 | 330.11 | 19.69 | 588.20 | 35.08 | 425.63 | 25.29 | 374.58 | 22.28 | 288.06 | 17.13 | 512.60 | 30.52 |
| 18:1-14:0 | 3078.72 | 200.92 | 2360.43 | 154.04 | 1814.28 | 118.40 | 3232.66 | 210.96 | 2331.91 | 152.07 | 2054.44 | 134.01 | 1579.29 | 103.01 | 2813.29 | 183.54 |
| 18:1-16:0 | 588.93 | 33.76 | 451.59 | 25.88 | 347.05 | 19.89 | 618.39 | 35.45 | 447.39 | 25.55 | 393.75 | 22.52 | 302.80 | 17.31 | 538.87 | 30.84 |
| 18:1-18:0 | 269.57 | 2.22 | 206.75 | 1.70 | 158.85 | 1.31 | 283.06 | 2.33 | 205.66 | 1.68 | 180.74 | 1.48 | 139.07 | 1.14 | 247.13 | 2.03 |

ASC-sEVs, small extracellular vesicles from human adipose tissue-derived mesenchymal stem cells

**Supplemental Table 7. Phosphatidic ethanolamine content in small extracellular vesicles (sEVs) originating from human adipose-derived mesenchymal stem cells and their donor cells (pmol/mg protein)**

|  | ASC-sEVs | | | | | | | | Donor Cells | | | | | | | |
| --- | --- | --- | --- | --- | --- | --- | --- | --- | --- | --- | --- | --- | --- | --- | --- | --- |
|  | Donor A | | Donor B | | Donor C | | Donor D | | Donor A | | Donor B | | Donor C | | Donor D | |
|  | Mean | SD | Mean | SD | Mean | SD | Mean | SD | Mean | SD | Mean | SD | Mean | SD | Mean | SD |
| 14:0 | 73.47 | 2.31 | 83.25 | 2.61 | 53.04 | 1.67 | 86.41 | 2.71 | 57.24 | 1.75 | 73.30 | 2.27 | 47.02 | 1.45 | 76.05 | 2.36 |
| 16:0 | 74.31 | 2.54 | 84.21 | 2.87 | 53.66 | 1.83 | 87.41 | 2.99 | 57.88 | 1.92 | 74.13 | 2.50 | 47.55 | 1.60 | 76.91 | 2.60 |
| 18:0 | 69.12 | 0.40 | 78.33 | 0.45 | 49.91 | 0.29 | 81.30 | 0.47 | 53.95 | 0.30 | 69.02 | 0.39 | 44.29 | 0.25 | 71.60 | 0.41 |
| 14:0-16:0 | 67.80 | 1.82 | 76.84 | 2.06 | 48.95 | 1.32 | 79.74 | 2.14 | 52.94 | 1.38 | 67.72 | 1.79 | 43.46 | 1.14 | 70.25 | 1.86 |
| 14:0-18:0 | 144.23 | 13.43 | 163.32 | 15.20 | 104.14 | 9.70 | 169.62 | 15.80 | 110.79 | 10.17 | 142.96 | 13.22 | 91.47 | 8.44 | 148.44 | 13.74 |
| 16:0-14:0 | 114.54 | 7.87 | 129.73 | 8.91 | 82.70 | 5.69 | 134.71 | 9.26 | 88.32 | 5.96 | 113.73 | 7.75 | 72.82 | 4.95 | 118.07 | 8.06 |
| 16:0-18:0 | 620.56 | 27.85 | 702.34 | 31.52 | 448.10 | 20.11 | 729.79 | 32.76 | 471.33 | 21.08 | 611.91 | 27.42 | 390.72 | 17.50 | 635.79 | 28.50 |
| 16:0-18:1 | 114.81 | 16.86 | 130.03 | 19.08 | 82.90 | 12.18 | 135.03 | 19.83 | 88.53 | 12.76 | 114.00 | 16.60 | 72.99 | 10.59 | 118.34 | 17.25 |
| 16:0-18:2 | 38.74 | 2.58 | 43.95 | 2.92 | 27.96 | 1.86 | 45.57 | 3.03 | 30.95 | 1.95 | 39.10 | 2.54 | 25.20 | 1.62 | 40.51 | 2.64 |
| 16:0-20:4 | 38.77 | 3.89 | 43.98 | 4.41 | 27.99 | 2.81 | 45.60 | 4.58 | 30.97 | 2.95 | 39.13 | 3.83 | 25.22 | 2.45 | 40.54 | 3.98 |
| 16:0-22:6 | 3111.85 | 38.09 | 3521.48 | 43.10 | 2247.06 | 27.50 | 3659.55 | 44.79 | 2356.99 | 28.83 | 3064.56 | 37.49 | 1955.81 | 23.93 | 3184.68 | 38.97 |
| 18:0-14:0 | 1346.25 | 53.89 | 1523.53 | 60.98 | 972.12 | 38.91 | 1583.20 | 63.38 | 1020.60 | 40.79 | 1326.34 | 53.05 | 846.61 | 33.86 | 1378.25 | 55.14 |
| 18:0-16:0 | 645.28 | 39.41 | 730.31 | 44.60 | 465.95 | 28.46 | 758.86 | 46.35 | 490.04 | 29.83 | 636.24 | 38.80 | 406.24 | 24.76 | 661.08 | 40.32 |
| 18:0-18:1 | 1175.53 | 59.53 | 1330.35 | 67.36 | 848.84 | 42.99 | 1382.44 | 70.00 | 891.39 | 45.06 | 1158.27 | 58.60 | 739.36 | 37.40 | 1203.59 | 60.90 |
| 18:0-18:2 | 4090.96 | 231.96 | 4629.44 | 262.49 | 2954.07 | 167.50 | 4810.98 | 272.79 | 3098.07 | 175.57 | 4028.48 | 228.37 | 2570.91 | 145.73 | 4186.42 | 237.33 |
| 18:0-20:4 | 2071.94 | 48.14 | 2344.72 | 54.48 | 1496.14 | 34.77 | 2436.62 | 56.62 | 1569.88 | 36.44 | 2040.78 | 47.40 | 1302.51 | 30.25 | 2120.73 | 49.26 |
| 18:0-22:6 | 158.40 | 14.98 | 179.36 | 16.95 | 114.38 | 10.82 | 186.29 | 17.62 | 121.52 | 11.34 | 156.92 | 14.75 | 100.38 | 9.41 | 162.95 | 15.33 |
| 18:1-14:0 | 1129.01 | 21.87 | 1277.70 | 24.75 | 815.25 | 15.80 | 1327.73 | 25.72 | 856.18 | 16.56 | 1112.47 | 21.53 | 710.14 | 13.74 | 1155.99 | 22.38 |
| 18:1-16:0 | 22.49 | 2.70 | 25.56 | 3.05 | 16.23 | 1.95 | 26.46 | 3.17 | 18.65 | 2.04 | 23.11 | 2.66 | 14.99 | 1.69 | 23.89 | 2.76 |
| 18:1-18:0 | 126.36 | 14.20 | 143.10 | 16.07 | 91.24 | 10.26 | 148.61 | 16.70 | 97.27 | 10.75 | 125.37 | 13.98 | 80.25 | 8.92 | 130.16 | 14.53 |

ASC-sEVs, small extracellular vesicles from human adipose tissue-derived mesenchymal stem cells

**Supplemental Table 8. Phosphatidic serine content in small extracellular vesicles (sEVs) originating from human adipose-derived mesenchymal stem cells and their donor cells (pmol/mg protein)**

|  | ASC-sEVs | | | | | | | | Donor Cells | | | | | | | |
| --- | --- | --- | --- | --- | --- | --- | --- | --- | --- | --- | --- | --- | --- | --- | --- | --- |
|  | Donor A | | Donor B | | Donor C | | Donor D | | Donor A | | Donor B | | Donor C | | Donor D | |
|  | Mean | SD | Mean | SD | Mean | SD | Mean | SD | Mean | SD | Mean | SD | Mean | SD | Mean | SD |
| 14:0 | 113.16 | 2.62 | 156.30 | 3.61 | 84.53 | 1.95 | 158.44 | 3.66 | 87.28 | 1.98 | 136.86 | 3.14 | 74.41 | 1.70 | 138.72 | 3.19 |
| 16:0 | 18.99 | 1.04 | 26.34 | 1.43 | 14.18 | 0.78 | 26.60 | 1.46 | 16.00 | 0.79 | 23.79 | 1.25 | 13.20 | 0.68 | 24.01 | 1.27 |
| 18:0 | 116.86 | 13.28 | 161.40 | 18.32 | 87.28 | 9.92 | 163.61 | 18.59 | 90.08 | 10.05 | 141.29 | 15.94 | 76.81 | 8.63 | 143.21 | 16.17 |
| 14:0-16:0 | 293.52 | 10.30 | 405.20 | 14.22 | 219.25 | 7.70 | 410.95 | 14.42 | 223.79 | 7.80 | 353.39 | 12.37 | 191.62 | 6.70 | 358.39 | 12.55 |
| 14:0-18:0 | 228.25 | 16.00 | 315.12 | 22.09 | 170.49 | 11.95 | 319.56 | 22.41 | 174.39 | 12.11 | 275.03 | 19.21 | 149.20 | 10.40 | 278.89 | 19.49 |
| 16:0-14:0 | 5.47 | 0.71 | 7.68 | 0.97 | 4.08 | 0.53 | 7.67 | 0.99 | 5.76 | 0.53 | 7.55 | 0.85 | 4.42 | 0.46 | 7.54 | 0.86 |
| 16:0-18:0 | 14.19 | 1.11 | 19.71 | 1.53 | 10.59 | 0.83 | 19.87 | 1.55 | 12.36 | 0.84 | 18.02 | 1.33 | 10.08 | 0.72 | 18.16 | 1.35 |
| 16:0-18:1 | 95.70 | 6.69 | 132.21 | 9.23 | 71.48 | 4.99 | 134.00 | 9.36 | 74.07 | 5.06 | 115.89 | 8.03 | 63.06 | 4.34 | 117.45 | 8.14 |
| 16:0-18:2 | 223.58 | 16.96 | 308.68 | 23.40 | 167.01 | 12.67 | 313.02 | 23.74 | 170.85 | 12.84 | 269.42 | 20.36 | 146.16 | 11.02 | 273.20 | 20.66 |
| 16:0-20:4 | 82.90 | 6.11 | 114.55 | 8.43 | 61.92 | 4.56 | 116.08 | 8.55 | 64.38 | 4.62 | 100.52 | 7.33 | 54.74 | 3.97 | 101.86 | 7.44 |
| 16:0-22:6 | 14.77 | 1.40 | 20.51 | 1.94 | 11.02 | 1.05 | 20.69 | 1.97 | 12.80 | 1.06 | 18.72 | 1.69 | 10.46 | 0.91 | 18.87 | 1.71 |
| 18:0-14:0 | 12.62 | 1.13 | 17.55 | 1.56 | 9.42 | 0.85 | 17.68 | 1.58 | 11.18 | 0.86 | 16.14 | 1.36 | 9.07 | 0.74 | 16.25 | 1.38 |
| 18:0-16:0 | 33.71 | 0.82 | 46.65 | 1.14 | 25.17 | 0.61 | 47.20 | 1.15 | 27.14 | 0.62 | 41.46 | 0.99 | 22.77 | 0.53 | 41.94 | 1.00 |
| 18:0-18:1 | 12.09 | 0.76 | 16.83 | 1.05 | 9.03 | 0.57 | 16.94 | 1.06 | 10.78 | 0.57 | 15.51 | 0.91 | 8.72 | 0.49 | 15.61 | 0.92 |
| 18:0-18:2 | 7.75 | 0.14 | 10.84 | 0.19 | 5.78 | 0.10 | 10.87 | 0.19 | 7.50 | 0.10 | 10.30 | 0.16 | 5.90 | 0.09 | 10.33 | 0.17 |
| 18:0-20:4 | 2.76 | 0.07 | 3.95 | 0.10 | 2.06 | 0.06 | 3.88 | 0.10 | 3.72 | 0.06 | 4.31 | 0.09 | 2.66 | 0.05 | 4.25 | 0.09 |
| 18:0-22:6 | 4.56 | 0.36 | 6.43 | 0.49 | 3.40 | 0.27 | 6.39 | 0.50 | 5.08 | 0.27 | 6.46 | 0.43 | 3.83 | 0.23 | 6.43 | 0.43 |
| 18:1-14:0 | 4.03 | 0.20 | 5.70 | 0.27 | 3.01 | 0.15 | 5.66 | 0.28 | 4.68 | 0.15 | 5.83 | 0.24 | 3.48 | 0.13 | 5.80 | 0.24 |
| 18:1-16:0 | 1.88 | 0.20 | 2.74 | 0.28 | 1.40 | 0.15 | 2.65 | 0.28 | 3.05 | 0.15 | 3.25 | 0.24 | 2.09 | 0.13 | 3.18 | 0.24 |
| 18:1-18:0 | 2.38 | 0.04 | 3.42 | 0.05 | 1.77 | 0.03 | 3.34 | 0.05 | 3.43 | 0.03 | 3.84 | 0.05 | 2.41 | 0.03 | 3.78 | 0.05 |

ASC-sEVs, small extracellular vesicles from human adipose tissue-derived mesenchymal stem cells

**Supplemental Table 9. Phosphatidic acid content in small extracellular vesicles (sEVs) originating from human adipose-derived mesenchymal stem cells and their donor cells (pmol/mg protein)**

|  | ASC-sEVs | | | | | | | | Donor Cells | | | | | | | |
| --- | --- | --- | --- | --- | --- | --- | --- | --- | --- | --- | --- | --- | --- | --- | --- | --- |
|  | Donor A | | Donor B | | Donor C | | Donor D | | Donor A | | Donor B | | Donor C | | Donor D | |
|  | Mean | SD | Mean | SD | Mean | SD | Mean | SD | Mean | SD | Mean | SD | Mean | SD | Mean | SD |
| 14:0 | 49.22 | 5.51 | 68.06 | 7.60 | 40.85 | 4.57 | 82.71 | 9.25 | 351.55 | 38.54 | 483.45 | 53.19 | 292.92 | 31.99 | 585.96 | 64.75 |
| 16:0 | 8.49 | 1.09 | 11.85 | 1.51 | 7.04 | 0.91 | 14.27 | 1.83 | 66.41 | 7.64 | 89.95 | 10.55 | 56.25 | 6.34 | 106.92 | 12.84 |
| 18:0 | 7.30 | 0.70 | 10.21 | 0.96 | 6.05 | 0.58 | 12.28 | 1.17 | 58.09 | 4.87 | 78.47 | 6.72 | 49.35 | 4.04 | 92.95 | 8.18 |
| 18:1 | 14.75 | 0.12 | 20.49 | 0.17 | 12.23 | 0.10 | 24.79 | 0.21 | 110.22 | 0.86 | 150.41 | 1.19 | 92.61 | 0.72 | 180.52 | 1.45 |
| 18:2 | 7.98 | 0.76 | 11.15 | 1.05 | 6.62 | 0.63 | 13.43 | 1.28 | 62.88 | 5.34 | 85.08 | 7.37 | 53.32 | 4.43 | 100.99 | 8.97 |
| 20:4 | 5.74 | 0.74 | 8.06 | 1.03 | 4.76 | 0.62 | 9.66 | 1.25 | 1.15 | 0.13 | 1.54 | 0.17 | 0.98 | 0.10 | 1.81 | 0.21 |
| 22:6 | 2.13 | 0.24 | 3.07 | 0.33 | 1.76 | 0.20 | 3.59 | 0.40 | 0.53 | 0.04 | 0.69 | 0.06 | 0.47 | 0.03 | 0.78 | 0.07 |
| 14:0-16:0 | 3.95 | 0.31 | 5.59 | 0.43 | 3.27 | 0.26 | 6.65 | 0.52 | 34.63 | 2.18 | 46.10 | 3.01 | 29.88 | 1.81 | 53.54 | 3.67 |
| 14:0-18:0 | 1.80 | 0.18 | 2.62 | 0.25 | 1.49 | 0.15 | 3.04 | 0.31 | 19.60 | 1.29 | 25.36 | 1.78 | 17.40 | 1.07 | 28.29 | 2.17 |
| 14:0-18:1 | 1.56 | 0.18 | 2.28 | 0.24 | 1.28 | 0.15 | 2.63 | 0.30 | 17.89 | 1.24 | 22.99 | 1.71 | 15.98 | 1.03 | 25.41 | 2.08 |
| 16:0-18:1 | 1.64 | 0.13 | 2.40 | 0.17 | 1.35 | 0.10 | 2.77 | 0.21 | 18.46 | 0.88 | 23.77 | 1.21 | 16.45 | 0.73 | 26.36 | 1.48 |
| 16:0-18:2 | 1.42 | 0.04 | 2.09 | 0.06 | 1.17 | 0.03 | 2.40 | 0.07 | 0.00 | 0.00 | 0.00 | 0.00 | 0.00 | 0.00 | 0.00 | 0.00 |
| 16:0-20:4 | 1.22 | 0.03 | 1.82 | 0.04 | 1.01 | 0.02 | 2.07 | 0.05 | 0.00 | 0.00 | 0.00 | 0.00 | 0.00 | 0.00 | 0.00 | 0.00 |
| 18:0-18:1 | 1.08 | 0.00 | 1.63 | 0.00 | 0.89 | 0.00 | 1.83 | 0.00 | 14.57 | 0.02 | 18.42 | 0.02 | 13.23 | 0.01 | 19.84 | 0.03 |
| 18:0-18:2 | 1.15 | 0.01 | 1.73 | 0.01 | 0.95 | 0.01 | 1.96 | 0.01 | 0.00 | 0.00 | 0.00 | 0.00 | 0.00 | 0.00 | 0.00 | 0.00 |
| 18:0-20:4 | 76.01 | 5.45 | 105.03 | 7.52 | 63.08 | 4.52 | 127.71 | 9.15 | 0.00 | 0.00 | 0.00 | 0.00 | 0.00 | 0.00 | 0.00 | 0.00 |
| 18:0-22:6 | 14.66 | 0.71 | 20.37 | 0.99 | 12.16 | 0.59 | 24.64 | 1.20 | 0.00 | 0.00 | 0.00 | 0.00 | 0.00 | 0.00 | 0.00 | 0.00 |

ASC-sEVs, small extracellular vesicles from human adipose tissue-derived mesenchymal stem cells

**Supplemental Table 10. Cholesterol and metabolites content in small extracellular vesicles (sEVs) originating from human adipose-derived mesenchymal stem cells and their donor cells (pmol/mg protein)**

|  | ASC-sEVs | | | | | | | | Donor Cells | | | | | | | |
| --- | --- | --- | --- | --- | --- | --- | --- | --- | --- | --- | --- | --- | --- | --- | --- | --- |
|  | Donor A | | Donor B | | Donor C | | Donor D | | Donor A | | Donor B | | Donor C | | Donor D | |
|  | Mean | SD | Mean | SD | Mean | SD | Mean | SD | Mean | SD | Mean | SD | Mean | SD | Mean | SD |
| Cholesterol | 41.65 | 0.91 | 41.15 | 0.90 | 31.38 | 0.69 | 69.99 | 1.54 | 51.34 | 1.08 | 45.76 | 0.98 | 35.15 | 0.75 | 77.06 | 1.67 |
| C18:1 cholesterol | 3.12 | 0.05 | 3.09 | 0.05 | 4.41 | 0.07 | 5.32 | 0.08 | 160.87 | 2.34 | 159.14 | 2.31 | 222.18 | 3.28 | 265.60 | 3.93 |
| C16:1 cholesterol | 2.14 | 0.04 | 2.12 | 0.04 | 3.03 | 0.05 | 3.67 | 0.06 | 60.48 | 0.89 | 59.92 | 0.88 | 82.83 | 1.25 | 98.86 | 1.50 |
| 27-hydroxy Cholesterol | 1.27 | 0.06 | 0.82 | 0.03 | 1.64 | 0.08 | 0.72 | 0.03 | 13.04 | 0.28 | 3.98 | 0.08 | 5.78 | 0.18 | 3.75 | 0.07 |
| 7α-hydroxy Cholesterol | 1.37 | 0.06 | 0.88 | 0.03 | 1.78 | 0.08 | 0.77 | 0.03 | 7.53 | 0.15 | 2.97 | 0.06 | 4.40 | 0.13 | 2.81 | 0.05 |
| 25-hydroxy Cholesterol | 2.67 | 0.04 | 1.65 | 0.03 | 3.60 | 0.06 | 1.50 | 0.02 | 19.70 | 0.21 | 5.80 | 0.06 | 10.05 | 0.14 | 5.46 | 0.05 |
| 6-keto-5α-hydroxycholesterol | 3.16 | 0.06 | 1.95 | 0.03 | 4.30 | 0.08 | 1.78 | 0.03 | 12.05 | 0.14 | 4.68 | 0.05 | 8.40 | 0.12 | 4.40 | 0.05 |
| 7-keto Cholesterol | 2.26 | 0.07 | 1.41 | 0.04 | 3.02 | 0.09 | 1.27 | 0.04 | 9.77 | 0.17 | 3.82 | 0.06 | 6.38 | 0.15 | 3.60 | 0.06 |

ASC-sEVs, small extracellular vesicles from human adipose tissue-derived mesenchymal stem cells

**Supplemental Table 11. Ceramide NS content in small extracellular vesicles (sEVs) originating from human adipose-derived mesenchymal stem cells and their donor cells (pmol/mg protein)**

|  | ASC-sEVs | | | | | | | | Donor Cells | | | | | | | |
| --- | --- | --- | --- | --- | --- | --- | --- | --- | --- | --- | --- | --- | --- | --- | --- | --- |
|  | Donor A | | Donor B | | Donor C | | Donor D | | Donor A | | Donor B | | Donor C | | Donor D | |
|  | Mean | SD | Mean | SD | Mean | SD | Mean | SD | Mean | SD | Mean | SD | Mean | SD | Mean | SD |
| C14 | 3.20 | 0.36 | 4.56 | 0.50 | 3.09 | 0.36 | 5.40 | 0.61 | 2.03 | 0.12 | 3.25 | 0.29 | 2.39 | 0.21 | 3.74 | 0.36 |
| C16 | 170.65 | 14.06 | 235.64 | 19.40 | 169.87 | 14.00 | 286.71 | 23.62 | 59.42 | 4.82 | 138.54 | 11.36 | 100.03 | 8.20 | 168.44 | 13.83 |
| C18 | 30.75 | 1.04 | 42.58 | 1.43 | 30.53 | 1.03 | 51.68 | 1.74 | 11.47 | 0.36 | 25.51 | 0.84 | 18.46 | 0.61 | 30.84 | 1.02 |
| C20 | 20.92 | 1.15 | 29.01 | 1.59 | 20.74 | 1.15 | 35.16 | 1.94 | 8.10 | 0.40 | 17.57 | 0.93 | 12.73 | 0.67 | 21.17 | 1.13 |
| C22 | 108.32 | 3.83 | 149.61 | 5.28 | 107.78 | 3.81 | 181.99 | 6.43 | 38.05 | 1.31 | 88.18 | 3.09 | 63.69 | 2.23 | 107.13 | 3.77 |
| C24:1 | 554.30 | 20.33 | 765.07 | 28.06 | 551.98 | 20.25 | 931.24 | 34.16 | 190.91 | 6.97 | 448.48 | 16.43 | 323.73 | 11.86 | 545.76 | 20.00 |
| C24 | 159.63 | 8.57 | 220.42 | 11.83 | 158.89 | 8.54 | 268.19 | 14.40 | 55.64 | 2.94 | 129.63 | 6.92 | 93.60 | 5.00 | 157.59 | 8.43 |
| C26:1 | 2.50 | 0.07 | 3.59 | 0.10 | 2.39 | 0.07 | 4.21 | 0.12 | 1.78 | 0.03 | 2.68 | 0.06 | 1.98 | 0.04 | 3.05 | 0.07 |
| C26 | 7.77 | 0.29 | 10.87 | 0.40 | 7.64 | 0.29 | 13.08 | 0.48 | 3.59 | 0.10 | 6.95 | 0.23 | 5.06 | 0.17 | 8.24 | 0.28 |
| Total | 1058.04 | 40.88 | 1461.34 | 56.41 | 1052.91 | 40.72 | 1777.66 | 68.68 | 370.98 | 14.01 | 860.79 | 33.03 | 621.68 | 23.84 | 1045.97 | 40.21 |

ASC-sEVs, small extracellular vesicles from human adipose tissue-derived mesenchymal stem cells

**Supplemental Table 12. Ceramide NDS content in small extracellular vesicles (sEVs) originating from human adipose-derived mesenchymal stem cells and their donor cells (pmol/mg protein)**

|  | ASC-sEVs | | | | | | | | Donor Cells | | | | | | | |
| --- | --- | --- | --- | --- | --- | --- | --- | --- | --- | --- | --- | --- | --- | --- | --- | --- |
|  | Donor A | | Donor B | | Donor C | | Donor D | | Donor A | | Donor B | | Donor C | | Donor D | |
|  | Mean | SD | Mean | SD | Mean | SD | Mean | SD | Mean | SD | Mean | SD | Mean | SD | Mean | SD |
| C14 | 0.10 | 0.00 | 0.27 | 0.01 | 0.07 | 0.00 | 0.12 | 0.00 | 0.14 | 0.00 | 0.22 | 0.00 | 0.11 | 0.00 | 0.14 | 0.00 |
| C16 | 159.44 | 12.66 | 220.16 | 17.46 | 132.32 | 10.50 | 184.74 | 14.66 | 54.75 | 4.34 | 128.95 | 10.22 | 77.53 | 6.15 | 108.22 | 8.58 |
| C18 | 0.37 | 0.01 | 0.65 | 0.01 | 0.30 | 0.01 | 0.44 | 0.01 | 0.23 | 0.00 | 0.45 | 0.01 | 0.24 | 0.01 | 0.32 | 0.01 |
| C20 | 0.27 | 0.03 | 0.50 | 0.03 | 0.21 | 0.02 | 0.32 | 0.03 | 0.19 | 0.01 | 0.36 | 0.02 | 0.19 | 0.01 | 0.25 | 0.02 |
| C22 | 1.10 | 0.12 | 1.65 | 0.16 | 0.90 | 0.10 | 1.28 | 0.13 | 0.48 | 0.04 | 1.03 | 0.09 | 0.59 | 0.06 | 0.82 | 0.08 |
| C24:1 | 72.90 | 2.64 | 100.75 | 3.65 | 60.50 | 2.19 | 84.48 | 3.06 | 25.09 | 0.91 | 59.04 | 2.14 | 35.48 | 1.28 | 49.52 | 1.79 |
| C24 | 21.60 | 1.11 | 29.94 | 1.54 | 17.92 | 0.92 | 25.03 | 1.29 | 7.50 | 0.38 | 17.59 | 0.90 | 10.55 | 0.54 | 14.72 | 0.76 |
| C26:1 | 0.09 | 0.00 | 0.26 | 0.00 | 0.07 | 0.00 | 0.12 | 0.00 | 0.13 | 0.00 | 0.22 | 0.00 | 0.10 | 0.00 | 0.13 | 0.00 |
| C26 | 0.14 | 0.01 | 0.33 | 0.02 | 0.10 | 0.01 | 0.17 | 0.02 | 0.15 | 0.00 | 0.26 | 0.01 | 0.13 | 0.01 | 0.16 | 0.01 |
| Total | 256.00 | 15.66 | 354.52 | 21.61 | 212.40 | 13.00 | 296.71 | 18.15 | 88.66 | 5.37 | 208.13 | 12.65 | 124.93 | 7.61 | 174.28 | 10.62 |

ASC-sEVs, small extracellular vesicles from human adipose tissue-derived mesenchymal stem cells

**Supplemental Table 13. Sphingomyelin content in small extracellular vesicles (sEVs) originating from human adipose-derived mesenchymal stem cells and their donor cells (pmol/mg protein)**

|  | ASC-sEVs | | | | | | | | Donor Cells | | | | | | | |
| --- | --- | --- | --- | --- | --- | --- | --- | --- | --- | --- | --- | --- | --- | --- | --- | --- |
|  | Donor A | | Donor B | | Donor C | | Donor D | | Donor A | | Donor B | | Donor C | | Donor D | |
|  | Mean | SD | Mean | SD | Mean | SD | Mean | SD | Mean | SD | Mean | SD | Mean | SD | Mean | SD |
| C14 | 13.98 | 1.68 | 23.31 | 2.78 | 19.71 | 2.37 | 26.31 | 3.16 | 7.72 | 0.79 | 16.66 | 1.91 | 14.19 | 1.62 | 18.72 | 2.17 |
| C16 | 511.73 | 16.67 | 847.60 | 27.61 | 722.04 | 23.52 | 962.90 | 31.37 | 241.58 | 7.83 | 581.66 | 18.92 | 495.60 | 16.12 | 660.69 | 21.50 |
| C18 | 124.05 | 11.82 | 205.59 | 19.57 | 175.01 | 16.67 | 233.42 | 22.23 | 59.43 | 5.55 | 141.60 | 13.41 | 120.65 | 11.43 | 160.68 | 15.24 |
| C20 | 144.40 | 3.53 | 239.29 | 5.85 | 203.73 | 4.98 | 271.72 | 6.65 | 69.00 | 1.66 | 164.70 | 4.01 | 140.33 | 3.42 | 186.93 | 4.56 |
| C22 | 570.58 | 34.36 | 945.05 | 56.90 | 805.08 | 48.48 | 1073.63 | 64.65 | 269.23 | 16.14 | 648.46 | 39.00 | 552.52 | 33.23 | 736.59 | 44.31 |
| C24:1 | 935.87 | 20.56 | 1549.97 | 34.04 | 1320.50 | 29.00 | 1760.95 | 38.68 | 440.85 | 9.66 | 1063.09 | 23.33 | 905.80 | 19.88 | 1207.71 | 26.51 |
| C24 | 468.17 | 25.65 | 775.45 | 42.48 | 660.57 | 36.20 | 880.93 | 48.27 | 221.11 | 12.05 | 532.21 | 29.12 | 453.47 | 24.81 | 604.50 | 33.09 |
| C26:1 | 3.08 | 0.64 | 5.27 | 1.06 | 4.33 | 0.90 | 5.82 | 1.20 | 2.60 | 0.30 | 4.30 | 0.73 | 3.66 | 0.62 | 4.67 | 0.83 |
| C26 | 1.30 | 0.09 | 2.32 | 0.16 | 1.82 | 0.13 | 2.46 | 0.18 | 1.77 | 0.04 | 2.27 | 0.11 | 1.93 | 0.09 | 2.37 | 0.12 |
| lyso sphingosylcholine | 10.63 | 0.24 | 17.77 | 0.39 | 14.98 | 0.34 | 20.02 | 0.45 | 107.24 | 1.67 | 207.96 | 4.04 | 179.34 | 3.45 | 231.11 | 4.60 |
| lyso DHsphingosylcholine | 0.33 | 0.01 | 0.71 | 0.02 | 0.45 | 0.02 | 0.64 | 0.02 | 34.66 | 0.08 | 32.63 | 0.20 | 29.95 | 0.17 | 31.89 | 0.23 |
| Total | 2784.12 | 16.92 | 4612.32 | 28.03 | 3928.24 | 23.88 | 5238.81 | 31.84 | 1455.19 | 6.44 | 3395.54 | 15.56 | 2897.42 | 13.26 | 3845.87 | 17.68 |

ASC-sEVs, small extracellular vesicles from human adipose tissue-derived mesenchymal stem cells

**Supplemental Table 14. Content of sphingoid bases and sphingoid bases-1-phosphates in small extracellular vesicles (sEVs) originating from human adipose-derived mesenchymal stem cells and their donor cells (pmol/mg protein)**

|  | ASC-sEVs | | | | | | | | Donor Cells | | | | | | | |
| --- | --- | --- | --- | --- | --- | --- | --- | --- | --- | --- | --- | --- | --- | --- | --- | --- |
|  | Donor A | | Donor B | | Donor C | | Donor D | | Donor A | | Donor B | | Donor C | | Donor D | |
|  | Mean | SD | Mean | SD | Mean | SD | Mean | SD | Mean | SD | Mean | SD | Mean | SD | Mean | SD |
| S1P | 13.12 | 1.15 | 18.25 | 1.59 | 10.91 | 0.96 | 22.16 | 1.93 | 9.50 | 0.71 | 15.12 | 1.25 | 9.35 | 0.75 | 18.19 | 1.52 |
| So | 42.44 | 3.78 | 58.71 | 5.21 | 35.24 | 3.14 | 71.42 | 6.35 | 40.52 | 3.44 | 56.98 | 4.98 | 34.59 | 2.99 | 69.11 | 6.06 |
| Sa | 13.78 | 1.06 | 19.16 | 1.47 | 11.46 | 0.88 | 23.28 | 1.79 | 11.65 | 0.78 | 17.22 | 1.25 | 10.64 | 0.75 | 20.74 | 1.53 |
| Sa1P | 0.87 | 0.06 | 1.34 | 0.08 | 0.74 | 0.05 | 1.59 | 0.09 | 2.35 | 0.04 | 2.08 | 0.07 | 1.54 | 0.04 | 2.29 | 0.08 |

ASC-sEVs, small extracellular vesicles from human adipose tissue-derived mesenchymal stem cells

**Supplemental Table 15. Activity of ceramide metabolic enzymes in small extracellular vesicles (sEVs) originating from human adipose-derived mesenchymal stem cells and their donor cells (pmol/mg protein)**

|  | ASC-sEVs | | | | | | | | Donor Cells | | | | | | | |
| --- | --- | --- | --- | --- | --- | --- | --- | --- | --- | --- | --- | --- | --- | --- | --- | --- |
|  | Donor A | | Donor B | | Donor C | | Donor D | | Donor A | | Donor B | | Donor C | | Donor D | |
|  | Mean | SD | Mean | SD | Mean | SD | Mean | SD | Mean | SD | Mean | SD | Mean | SD | Mean | SD |
| SPT | 28.77 | 1.84 | 44.89 | 3.98 | 24.62 | 1.22 | 34.20 | 1.90 | 50.66 | 3.26 | 68.06 | 3.78 | 38.68 | 2.49 | 58.77 | 1.94 |
| CerS1 | 18.88 | 0.48 | 22.06 | 0.84 | 15.69 | 0.74 | 20.07 | 1.04 | 18.96 | 0.92 | 22.41 | 1.51 | 30.46 | 1.50 | 38.96 | 0.99 |
| CerS2 | 28.77 | 1.84 | 44.89 | 3.98 | 24.62 | 1.22 | 34.20 | 1.90 | 31.66 | 2.04 | 42.54 | 2.36 | 24.18 | 1.56 | 36.73 | 1.21 |
| CerS3 | 34.90 | 1.82 | 79.26 | 5.00 | 46.18 | 2.55 | 60.91 | 3.84 | 58.36 | 2.23 | 129.30 | 6.14 | 72.31 | 3.57 | 111.09 | 2.82 |
| CerS4 | 9.41 | 0.49 | 7.93 | 0.20 | 12.43 | 0.47 | 9.03 | 0.42 | 11.98 | 0.75 | 11.47 | 0.32 | 13.48 | 0.66 | 11.96 | 0.81 |
| CerS5/6 | 19.87 | 0.50 | 18.66 | 1.17 | 21.79 | 1.20 | 19.35 | 1.21 | 38.31 | 0.98 | 33.78 | 1.29 | 48.59 | 2.30 | 41.17 | 2.03 |
| Acidic CDase | 37.72 | 2.37 | 30.16 | 0.85 | 45.36 | 2.22 | 28.44 | 1.92 | 74.64 | 1.91 | 58.92 | 2.25 | 76.40 | 3.62 | 53.79 | 2.65 |
| Neutral CDase | 27.29 | 1.42 | 25.43 | 0.64 | 25.59 | 1.61 | 24.56 | 1.35 | 104.25 | 6.57 | 102.21 | 2.90 | 95.33 | 4.66 | 97.28 | 6.60 |
| Alkaline CDase | n.d. | n.d. | n.d. | n.d. | n.d. | n.d. | n.d. | n.d. | 34.24 | 0.87 | 26.95 | 1.69 | 35.73 | 1.97 | 26.73 | 0.68 |
| Acidic SMase | 102.51 | 6.46 | 120.04 | 3.40 | 96.54 | 4.72 | 129.00 | 8.75 | 84.41 | 2.16 | 96.83 | 3.71 | 81.88 | 3.89 | 111.19 | 5.49 |
| Neutral SMase | 39.35 | 1.00 | 46.47 | 2.93 | 37.23 | 2.05 | 52.09 | 1.33 | 43.85 | 1.24 | 51.25 | 2.51 | 45.77 | 3.10 | 59.25 | 3.09 |
| SM deacylase | 3.30 | 0.18 | 2.55 | 0.06 | 2.79 | 0.10 | 2.96 | 0.14 | 39.83 | 1.13 | 27.80 | 1.36 | 33.07 | 2.24 | 35.90 | 1.87 |
| SPHK1 | 6.30 | 0.34 | 7.68 | 0.19 | 5.88 | 0.22 | 7.41 | 0.35 | 5.08 | 0.14 | 6.92 | 0.33 | 4.64 | 0.31 | 6.10 | 0.31 |
| SPHK2 | n.d. | n.d. | n.d. | n.d. | n.d. | n.d. | n.d. | n.d. | 0.89 | 0.02 | 0.74 | 0.03 | 0.99 | 0.06 | 0.94 | 0.04 |
| S1P lyase | 0.13 | 0.00 | 0.12 | 0.00 | 0.13 | 0.00 | 0.12 | 0.00 | 36.07 | 1.02 | 31.92 | 1.56 | 40.77 | 2.76 | 36.84 | 1.92 |
| S1P Phosphatase | 1.33 | 0.03 | 1.11 | 0.06 | 1.42 | 0.07 | 1.17 | 0.03 | 82.04 | 2.32 | 70.17 | 3.43 | 85.90 | 5.82 | 75.08 | 3.92 |

ASC-sEVs, small extracellular vesicles from human adipose tissue-derived mesenchymal stem cells; SPT, serine palmitoyltransferase; CerS, ceramide synthase; CDase, ceramidase; SMase, sphingomyelinase; SPHK, sphingosine kinase; S1P, sphingosine-1-phosphate
